# *Candida* sp. Infections in Patients with Diabetes Mellitus

**DOI:** 10.3390/jcm8010076

**Published:** 2019-01-10

**Authors:** Célia F. Rodrigues, Maria Elisa Rodrigues, Mariana Henriques

**Affiliations:** 1 CEB, Centre of Biological Engineering, LIBRO-Laboratório de Investigação em Biofilmes Rosário Oliveira, University of Minho, 4710-057 Braga, Portugal; elisarodrigues@deb.uminho.pt (M.E.R.); mcrh@deb.uminho.pt (M.H.); 2 LEPABE—Department of Chemical Engineering, Faculty of Engineering, University of Porto, Rua Dr. Roberto Frias, s/n, 4200-465 Porto, Portugal

**Keywords:** *Candida*, biofilms, diabetes, medical devices, candidiasis, metabolic disorder, hyperglycemia, infection

## Abstract

Candidiasis has increased substantially worldwide over recent decades and is a significant cause of morbidity and mortality, especially among critically ill patients. Diabetes mellitus (DM) is a metabolic disorder that predisposes individuals to fungal infections, including those related to *Candida* sp., due to a immunosuppressive effect on the patient. This review aims to discuss the latest studies regarding the occurrence of candidiasis on DM patients and the pathophysiology and etiology associated with these co-morbidities. A comprehensive review of the literature was undertaken. PubMed, Scopus, Elsevier’s ScienceDirect, and Springer’s SpringerLink databases were searched using well-defined search terms. Predefined inclusion and exclusion criteria were applied to classify relevant manuscripts. Results of the review show that DM patients have an increased susceptibility to *Candida* sp. infections which aggravates in the cases of uncontrolled hyperglycemia. The conclusion is that, for these patients, the hospitalization periods have increased and are commonly associated with the prolonged use of indwelling medical devices, which also increase the costs associated with disease management.

## 1. Introduction

**Figure 1:**
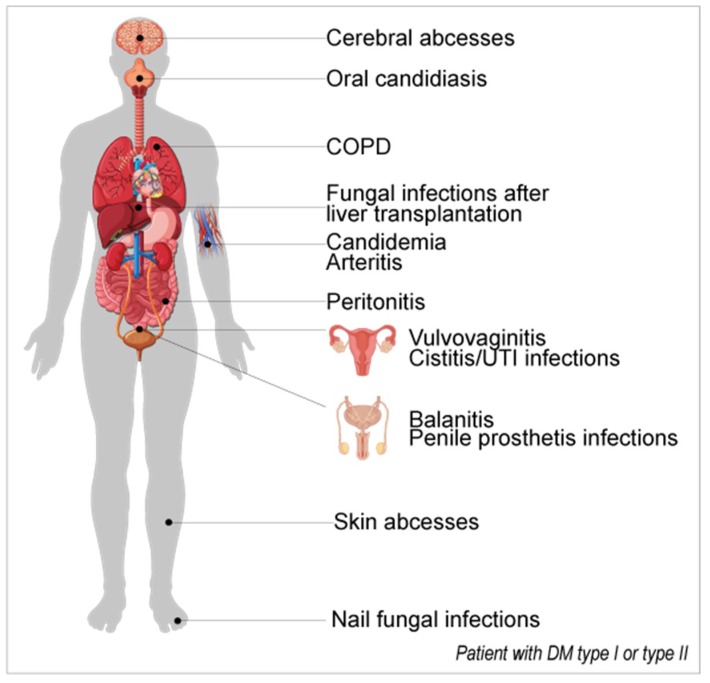
Main diseases related to *Candida* sp. occurring with higher incidence in patients with diabetes mellitus type 1 or type 2 (adapted image from GraphicsRF on stock.adobe.com).

Diabetes mellitus (DM) is a chronic metabolic and degenerative disorder that is characterized by chronic hyperglycemia and causes long-term complications like retinopathy, neuropathy, and nephropathy, generally accelerating macro- and micro-vascular changes. It is becoming one of the largest emerging threats to public health in the 21st century [1,2]. Several immune alterations have been described in diabetes with cellular immunity being more compromised and with changes in polymorphonuclear cells, monocytes, and lymphocytes [3]. DM individuals have higher glucose serum concentrations than healthy individuals (between 4.0 to 5.4 mmol/L or 72 to 99 mg/dL when fasting and up to 7.8 mmol/L or 140 mg/dL two hours after eating [4]; hemoglobin A1c (glycohemoglobin) ≤5.7%). In type 1 DM, the pathogenesis is multifactorial because of antibody-mediated autoimmunity, environmental toxins exposure, and major histocompatibility complex (MHC) Class II histocompatibility complex HLA-DR/DQ genetic polymorphisms. These features create an increased susceptibility to disease onset due to a continuous loss of insulin-producing β-cells in the pancreas, which is due to the T-cells’ infiltration through mitochondrial-driven apoptosis [5]. On the other hand, in type 2 DM, there is an insulin resistance that is associated with changes in the mitochondrial metabolism with reduced mitochondrial density, ATP production, and mitochondrial RNA (mtRNA) levels, as well as increased markers for oxidative stress. The chronic exposure of the circular mtDNA to these effects might trigger significant tissue modifications found in the pancreas and endothelial cells, leading to secondary vascular disease and causing cardiac, renal, ophthalmic, and neurological complications [5,6,7] (Figure 1).

**Figure 2:**
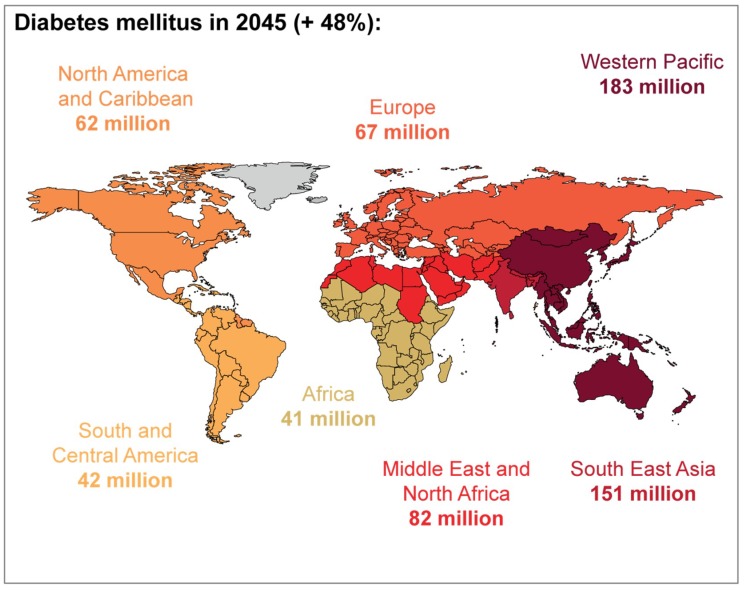
The estimated number of people with diabetes worldwide and per region in 2045 between 20–79 years in age, with a total of 629 million (source: International Diabetes Federation) (adapted image from GraphicsRF on stock.adobe.com).

In 2017, the worldwide prevalence of adult-onset diabetes (20–79 years) was nearly 425 million, and the World Health Organization and the International Diabetes Federation predicted that the number of adults in the world with diabetes will rise near 629 million by the year 2045 [8,9] (Figure 2). A higher prevalence of DM, cardiac, and pulmonary diseases can be found in senior patients with candidemia [10,11,12,13]. The relationship between diabetes and candidiasis has been widely studied [13,14,15,16], particularly due to the increased susceptibility of diabetic patients to fungal infections compared to those without DM [14,15,17,18].

Several mechanisms are attributed to higher *Candida* sp. predisposition among DM patients depending on the local or systemic infection. Among the recognized host conditions for candidal colonization and subsequent infection are yeast adhesion to epithelial cell surfaces [19], higher salivary glucose levels [15,20], reduced salivary flow [21], microvascular degeneration, and impaired candidacidal activity of neutrophils. These conditions are particularly serious in the presence of glucose [22,23], secretion of several degradative enzymes [24,25,26], or even a generalized immunosuppression state of the patient [8,27,28,29,30,31]. These factors have a major influence on the balance between host and yeasts, favoring the transition of *Candida* sp. from commensal to pathogen and causing infection. In fact, in a very recent study, Gürsoy et al. [32] suggested that there is a higher presence of intestinal *Candida albicans* colonization in diabetic patients. In fact, there may be a tendency of type 1 DM in patients with a high prevalence of intestinal *C. albicans*. *C. albicans* is also known to wait for a change in some aspect of the host physiology that normally suppress growth and invasiveness through a phenomenon called phenotypic switch system or white-opaque transition, described in 1987.

This involves reversible and heritable switching between alternative cellular phenotypes. It occurs at sites of infection and recurrently in episodes of infection in certain cases in diabetic patients [33].

Yeasts are part of the normal gut microflora, but cell counts do not normally surpass 10 colony forming units (CFU)/g feces [34,35]. Nevertheless, it has been described that *Candida* sp. is more widespread in the feces of patients with type 1 and type 2 DM with poor glycemic control as opposed to healthy subjects [36]. The main reasons for this colonization seem to be altered functions of the immune system in diabetic patients with poor glycemic control or a direct effect of elevated blood glucose levels, creating specific conditions for intensive fungal colonization [36]. In fact, another report [37] showed that in patients with type 1 DM, the total gut CFUs significantly rise up to 40% in *C. albicans* colonization compared to 14.3% in healthy individuals. This may be related to the decrease in commensal bacteria-probably the result of yeast-bacterial competition. Also, this higher growth may disrupt the ecological balance of intestinal flora, which occurs in type 1 DM [37]. Regarding the gastrointestinal colonization, Kowalewska et al. [38] studied the serum levels of interleukin-12 (IL12) in relation to the percentage of yeast-like fungi colonies residing in the gastrointestinal tract in children and adolescents with DM type 1. Results showed that high IL12 levels can inhibit infection with yeast-like fungal colonizing the gastrointestinal tract in children and adolescents with type 1 DM. However, further studies are needed to confirm the antifungal activity of IL12 [38].

The development of drug resistance among *Candida* sp. isolates allied to epidemiologic variations in *Candida* sp. natural flora has significant implications for morbidity and mortality [39,40,41,42]. The extensive use of medications, especially azoles, has promoted the selection of resistant species by shifting colonization to more naturally resistant *Candida* sp., such as *C*. *glabrata*, *C. dubliniensis*, and *C. krusei* [43,44,45,46]. Presently, the world distribution of *Candida* sp. is a feature of the epidemiology in the area, but it indicates a predominance of *C. albicans*, *C. glabrata*, *C. tropicalis*, *C. parapsilosis*, and *C. krusei* [45,47]. It has been confirmed that 90% of fungemia cases are attributed to *Candida* sp. [39,40], and the mortality has ranged from 40% to 80% in immunocompromised hosts [39,40,48]. Furthermore, a high mortality rate was also detected among non-immunocompromised patients (60%) [49] and those with diabetes (67%) [41].

The main pathophysiologic and nutritionally relevant sugars in diabetic patients are glucose and fructose, but other simple carbon sources also play an important part in the growth of *Candida* sp. in DM patients. Man et al. [50] evaluated the growth rate of *C. albicans* in the presence of different concentrations of glucose and fructose to obtain a better understanding of the nutrient acquisition strategy and its possible relation to the hyperglycemic status of diabetic patients. The authors determined that the glucose concentration is directly related to *C. albicans* growth, which may be linked to the frequent yeast infections that occur in non-controlled diabetic patients. Interestingly, fructose showed *C. albicans* inhibition capacities. This implies fructose-containing food may prevent the development of candidiasis. This is an important outcome in oral *Candida* sp. biofilms, especially for patients who use prosthesis [50]. In fact, other carbon compounds such as sucrose, maltose, and lactose increase the fungal population density [43,51,52] and decrease the activity of antifungal agents. A recent report explored the effects of glucose in diabetic mice on the susceptibility of *Candida* sp. to antifungal agents [53]. In that work, Mandal et al. [53] revealed that voriconazole (Vcz) has the greatest reduction in antifungal drug efficacy followed by amphotericin B (AmB). Glucose displayed a higher affinity to bind to Vcz through hydrogen bonding, decreasing the susceptibility of antifungal agents during chemotherapy. Additionally, Mandal et al. [53] confirmed that Vcz presented three important hydrogen bonds and AmB presented two hydrogen bonds that stabilized the glucose. In vivo results of the same study proposed that the physiologically relevant higher glucose level in the bloodstream of mice with DM might interact with the available selective agents during antifungal therapy, decreasing glucose activity by complex formation. Vcz-glucose and AmB-glucose complexes seem to present less effectiveness as their pure molecule. Accordingly, a proper selection of drugs for DM patients is important if we are to control infectious diseases [53]. Similarly, Rodaki et al. [54] studied the impact of glucose on *C. albicans* transcriptome for the modulation of carbon assimilatory pathways during pathogenesis. The elevated resistance to oxidative and cationic stresses and resistance to miconazole uncovered that glucose concentrations in the bloodstream have a significant impact upon *C. albicans* gene regulation. No significant susceptibility level was perceived for anidulafungin, while Vcz and AmB became less effective [54]. In another study, Rodrigues et al. [52] demonstrated that *C. glabrata* decreases its susceptibility to fluconazole when cultured in a medium enriched with glucose [52].

Accordingly, the aim of this review is to analyze the literature related to the occurrence of candidiasis in diabetic patients by discussing specific features of *Candida* sp. that relate directly to the occurrence of candidiasis in DM patients and related diseases, as well as by reviewing recent and relevant studies on the topic.

## 2. Particular Features of *Candida* sp. that Increase the Incidence of Candidiasis in Diabetic Patients

### 2.1. Enzymatic Activity

Several studies have established an association between hydrolytic enzymes activity and an increase in the pathogenic ability of *Candida* sp. [55,56].

It has been demonstrated that, due to higher blood glucose concentration, diabetic *Candida* sp. isolates present significantly higher hemolytic and esterase enzymatic activity, which may contribute to increased enzyme activity among diabetic patients [57,58,59,60]. The same authors also hypothesized that these species are more pathogenic under abnormal conditions such as DM [61,62]. Secreted aspartyl proteinases (SAP) capable of degrading numerous substrates that constitute host proteins in the oral cavity have also been studied. These enzymes are thought to help *Candida* sp. to acquire essential nitrogen for growth, to attach to and penetrate oral mucosa, or both [63,64]. They can also cause amplified vascular permeability, leading to inflammatory reactions [65] and clinical symptoms, which may disturb the humoral host defense [66]. Similarly, phospholipase (PL) targets the membrane phospholipids and digests these components, initiating cell lysis and facilitating the penetration of the infecting fungi [67]. This enzyme induces the accumulation of inflammatory cells and plasma proteins, releasing several inflammatory mediators in vivo [67].

Very recently, it was revealed that *C. albicans* hyphae induce both epithelial damage and innate immunity through the secretion of a cytolytic peptide toxin called candidalysin [68,69]. This enzyme is encoded by the hypha-associated *ECE1* gene and is the first peptide toxin to be identified in any human fungal pathogen. Candidalysin induces calcium ion influx and lactate dehydrogenase (LDH) release in oral epithelial cells, which are features of cell damage and membrane destabilization. Importantly, the study also reported that *C. albicans* mutants where the entire *ECE1* gene or the candidalysin-encoding region had been deleted have full invasive potential in vitro but are incapable of inducing tissue damage or cytokine release and are highly weakened in a mouse model of oropharyngeal candidiasis and a zebrafish swim bladder mucosal model [68].

### 2.2. Biofilm Formation

Biofilms are communities of microorganisms embedded in an extracellular matrix [70,71], which confer substantial resistance to antifungal therapy and increased host immune responses [72,73]. These communities can be formed in both biotic (e.g., mouth mucosae) or abiotic (e.g., catheters) surfaces [74,75]. In fact, candidemia are the most prevalent invasive mycoses worldwide with mortality rates close to 40% [76]. *Candida* sp. are often recognized as the origin of candidemia, urinary tract infections, and hospital pneumonia. In practically all of these cases, the infections are related with the use of a medical device and biofilm formation on its surface [20]. The most frequently colonized medical device is the central venous catheter used for administration of fluids, nutrients, and medicines [77]. The contamination of the catheter or the infusion fluid can arise from the skin of the patient, the hands of health professionals [77], or by migration into the catheter from a pre-existing lesion. Less commonly, if *Candida* sp. that colonize the gastrointestinal tract as a commensal start to develop a pathogenic behavior, they are able to infiltrate the intestinal mucosa and diffuse through the bloodstream. Consequently, circulating yeast may colonize the catheter endogenously. This is more common in cancer patients, as chemotherapy leads to damage to the intestinal mucosa [78]. In the other patients, infected catheters are the most significant source of bloodstream infections, followed by widespread invasive candidiasis. The catheter removal is recommended in patients with disseminated *Candida* sp. infection to enable disinfection of the blood and to increase prognosis [79,80].

**Figure 3:**
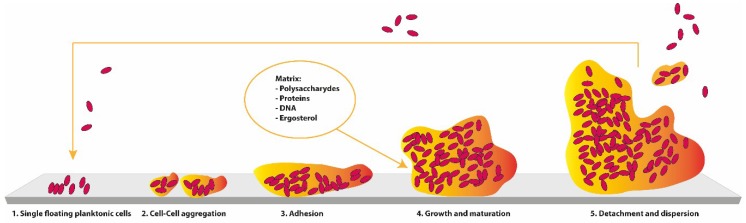
Development of a *Candida* sp. biofilm in a surface.

Biofilm development of *Candida* sp. (Figure 3) can be explained in four chronological steps: adherence-initial phase in which the yeast in suspension and planktonic cells adhere to the surface (first 1–3 h); intermediate phase-development of biofilm (11–14 h); maturation phase-the polymeric matrix (PEM) completely penetrates all layers of the cells adhered to the surface in a three-dimensional structure (20–48 h); dispersion-the most superficial cells leave the biofilm and colonize areas surrounding the surface (after 24 h) [81]. Hence, a mature biofilm comprises of a dense network of cells in the form of yeasts, hyphae, or pseudohyphae (or not, depending on the *Candida* sp.) involved by PEM and with water channels between the cells. These help in the diffusion of nutrients from the environment through the biomass to the lower layers and also allow the removal of waste [81,82]. Formed using in vivo models, *Candida* sp. biofilms seem to follow the same sequence of in vitro formation [83]. Nonetheless, the maturation step happens more quickly, and the thickness is increased. The final architecture of the biofilm is variable and depends, in part, on the *Candida* sp. involved, the growing conditions, and the substrate on which it is formed [81,84].

High levels of glucose are thought to serve as the carbohydrate energy source necessitated by *Candida* sp. for the biofilm formation and are probably required to produce the polysaccharide matrix [85], which is secreted by sessile cells, providing protection against environmental challenges [86]. Biofilm formation has been shown to be dependent on the *Candida* sp. and its clinical origin. Biofilms are refractory to antifungal drugs and more difficult to treat than infections with planktonic cells [44]. Moreover, it has been verified that *Candida* sp. isolated from patients with DM have a higher pathogenic potential for biofilm-forming [87]. The communities are extremely common on medical devices.

### 2.3. Hydrophobicity

In *Candida* sp., the adhesion phenomenon is mediated by agglutinin-like (Als) sequence proteins [88], which are glycosylphosphatidylinositol (GPI)-linked to β-1-6 glucans in the fungal cell wall. Als-dependent cellular adhesion is connected with increases in cell surface hydrophobicity (CSH) [89]. The CSH of *Candida* sp. enhances virulence by promoting adhesion to host tissues [89,90]. *C. albicans* Als3p (hypha-associated) is a major epithelial adhesin that is strongly upregulated during epithelial infection in vitro [90], and the disruption of the *ALS3* gene reduces epithelial adhesion in vitro. Likewise, decreasing the expression of the *ALS2* gene also reduces adhesion [91,92]. On the other side, deletion of the ALS5, ALS6, or ALS7 genes increased adhesion [93], demonstrating that the Als proteins can have opposing roles in fungal attachment to surfaces. Putative homologues of Als proteins have also been identified in NCAC [94]. In *C. glabrata*, for example, epithelial adhesins (Epa) have a comparable structure to the Als proteins [95,96].

Together with adhesion ability, hydrophobicity is a virulent factor that is gene-regulated and usually positively correlated with biofilm metabolic activity [97,98] since hydrophobic interactions seem to be crucial in promoting tissue invasion by the mycelial phase of *Candida* sp. It is presumed that *Candida* sp. can grow under anaerobic conditions, although in these conditions fermentation is the dominant pathway for ATP production [99]. The results of Sardi et al. [100] indicated that 51.97% of diabetic patients’ isolates were highly hydrophobic under anaerobic conditions when compared to 21.90% under the aerobic atmosphere [100]. It is recognized that the germ tubes are able to adhere to fibronectin, fibrinogen, and complement via cell surface receptors [101], helping the attachment of filament yeasts to extracellular matrix components (ECM) [102] and producing impairment of phagocytosis, consequently increasing the resistance to blood clearance and the virulence of *Candida* sp. [103].

## 3. Candidiasis and DM

### 3.1. Oral Diseases

#### 3.1.1. Oral Candidiasis

The prevalence of oral candidiasis is increasing, as it is one of the most common fungal infections [104]. Oral candidiasis can be diagnosed by the differential patterns of mucosal changes like erythematous, pseudomembranous, and curd-like plaques (biofilms) [105,106]. Higher *Candida* sp. colonization rates were reported in patients with DM type 1 when compared to DM type 2 patients (84% vs. 68%, respectively), while the percentage in nondiabetic subjects was around 27% [18]. The studies also describe how this colonization does not always lead to infection. Nonetheless, it is a prelude to infection when host immunity is compromised and the risk of a disseminated infection is high [107]. Such infections continue to be a major healthcare challenge [108].

The risk factors for oral candidal infection are complex, but it is known that tongue lesions, tobacco smoking, denture wearing, and immunosuppression (e.g., diabetes mellitus) clearly influence oral *Candida* sp. carriage and the upsurge of oral candidiasis [30,109,110,111,112,113,114]. The causes influencing the higher incidence of oral candidiasis in diabetic patients are presented in Table 1.

Higher expressions in enzymatic activity and the biofilm forming capacity of *Candida* sp. are two of the most important features in oral candidiasis. In a study by Sanitá et al. [115], the virulence of 148 clinical isolates of *C. albicans* from oral candidiasis was characterized by measuring the expression of PL and SAP in healthy subjects (HS), diabetics with oral candidiasis (DOC), and non-diabetics with oral candidiasis (NDOC). For PL, *C. albicans* from NDOC and DOC had the highest enzymatic activity (76.6%); for SAP, *C. albicans* from NDOC exhibited lower enzymatic activity (48.9%). Similar results have been reported in the past [59,87], with percentages greater than 90% for both PL and SAP activity among clinical isolates of *C. albicans* [26,116,117] found. Arlsan and colleagues found 12 different genotypes and compared the virulence factors of several *Candida* sp. isolated from the oral cavities of 142 healthy and diabetic individuals, with and without caries. Although the most isolated species was also *C. albicans*, there were statistical differences in terms of isolated *Candida* frequency between healthy subjects and diabetic patients. DM showed no effect on the activities of virulence factors (biofilm production, proteinase, and phospholipase activity) of *Candida* sp. Yet, different genotypes of *C. albicans* exhibited different virulence activities [118]. Other authors showed that the activity of SAPs suggestively rises in denture wearers with signs of candidiasis compared to denture wearers with a normal palatal mucosa [119]. The inconsistency of results of these reports can be explained by the large variation of intra-and interspecies of *Candida* and deviations in the methodology in most reports [24,26,59,87,116,117,119,120,121,122,123]. A longitudinal study by Sanchés-Vargas et al. [124] quantified biofilms in oral clinical isolates of *Candida* sp. from adults with local and systemic predisposing factors for candidiasis. Between the isolates, authors found *C. albicans*, *C. tropicalis*, *C. glabrata*, *C. krusei*, *C. lusitaniae*, *C. kefyr*, *C. guilliermondii*, and *C. pulcherrima* from the oral mucosa of totally and partially edentulous patients (62.3%) and the oral mucosa of diabetics (37.7%). On average, the oral isolates of *C. glabrata* were considered strong biofilm producers, whereas *C. albicans* (the most common species) and *C. tropicalis* were moderate producers. This might be because *C. glabrata* has been shown to have a higher aggressiveness, producing a great quantity of biofilm matrices yet also increasing chitin concentrations in the cell walls [125,126,127].

Additional important features are the oral pH and the glycemic control. A study performed by Samaranayake et al. [128] demonstrated that pyruvates and acetates are the major ionic species, generating a quick decrease in pH with *Candida* sp., as found in batch cultures of mixed saliva supplemented with glucose [128].

Other authors indicated that yeasts have a superior ability to adhere to epithelia and denture acrylic surfaces at a low pH of approximately 2–4.14 [31,129]. Balan and colleagues stated that during hyperglycemic episodes, the environmental alteration in the oral cavity increased salivary glucose and acid production, which favored the transition of *Candida* sp. from commensal to pathogen [31]. In another report, while comparing diabetics and non-diabetics, Pallavan et al. [130] verified that 70% of the healthy individuals had lower colonization and 43.3% of the diabetic patients had severe colonization by *Candida* sp., which was also observed in other studies [14,18,21,131]. Prediabetes is a condition where there is an elevation of plasma glucose above the normal range but below that of clinical diabetes [132]. Javed and their colleagues isolated oral *Candida* sp. from 100% of patients with prediabetes and from 65.7% of the controls, observing that the carriage of *C. albicans* was greater among patients with prediabetes (48.7%) than with controls (25.7%) [133]. They also observed that the colonization with *Candida* sp. reduced the salivary flow rate and was independent of glycemic status in patients with prediabetes [132].

In fact, while some studies found a direct significant association between glycosylated hemoglobin and oral *Candida* colonization [134,135], other authors found no relationship between glycosylated hemoglobin and high *Candida*-burden in patients with DM [14,18,136,137]. Furthermore, studies indicate that the concentration of glucose present in the gingival crevicular fluid is related to the blood glucose level [138]. The quality of glycemic control can also partially explain the presence of a significant relationship between subgingival plaque candidal colonization and higher concentrations of glucose [138].

Although most of the scientific community believes that diabetes is a risk factor associated with oral yeast infections, in a recent paper, Costa and colleagues [139] reported that the presence of yeasts in the oral cavity of patients with type 1 DM (60% of total) was not affected by diabetes, metabolic control, duration of the disease, salivary flow rate, or saliva buffer capacity by age, sex, place of residence, number of daily meals, consumption of sweets, or frequency of tooth brushing. *Candida albicans* was the most prevalent yeast species, but a higher number of yeast species was isolated in nondiabetics [139]. The fact that this study was developed exclusively in children may be related to this conclusion.

#### 3.1.2. Antifungal Treatment of Oral Candidiasis

Importantly, several reports have stated the importance of the evaluation of the susceptibility of the oral isolates to the antifungal drugs in order to choose proper therapy in diabetic patients to control the fungal diseases. Aitken-Saavedra et al. [160] revealed that 66% of the yeasts isolated in diabetic patients were *C. albicans*, followed by *C. glabrata* (20.7%). In patients with decompensated type 2 DM, higher levels of salivary acidification and a greater diversity and quantity of yeasts of the genus *Candida* were observed. When nystatin was administered in these patients, higher inhibition was observed at a lower pH [160]. The study presented by Lydia Rajakumari and Saravana Kumari [161] showed a lower glycemic control leads to a higher candidal colonization in diabetic patients. The predominant species was *C. albicans*, but among denture wearers, *C. glabrata* was predominant. More importantly, ketoconazole, fluconazole, and itraconazole were effective against the isolated *Candida* sp. [161].

Similarly, Premkumar et al. [162] stated that, although *C. albicans* was the most predominantly isolated species, *C. dubliniensis*, *C. tropicalis*, and *C. parapsilosis* were also observed. The authors showed variable resistance toward amphotericin B, and fluconazole was observed in clinical isolates from diabetics but not from healthy patients. Again, a positive correlation was observed between glycemic control and candidal colonization [162]. In 2011, Bremenkamp et al. [163] found no significant differences in antifungal susceptibility to the tested agents between *Candida* sp. isolates from diabetic and non-diabetic subjects, which was consistent with the study by Manfredi et al. [164]. Furthermore, a high prevalence of *C. dubliniensis* in diabetic patients was found, which may suggest a potential misdiagnosis of its morphologically-related species, *C. albicans*. Other authors found the same two species in DM patients [1,16,57,135]. In yet another study, Sanitá and their colleagues [165] investigated the susceptibility of 198 oral clinical isolates of *Candida* sp. against caspofungin, amphotericin B, itraconazole, and fluconazole. Their findings confirmed the resistant profile of *C. glabrata* isolates against azole antifungals—especially itraconazole—in individuals with diabetes and denture stomatitis. The clinical sources of the isolates were shown to have no effect on the mininum inhibitory concentration (MIC) values obtained for all antifungals tested, which was in accordance with previous reports [26,119]. Additionally, *Candida* sp. isolates with higher rates of resistance to flucytosine, ketoconazole, miconazole, and econazole were confirmed in patients with diabetes when compared to healthy controls [163,166]. The increase in the environment glucose concentration may trigger the expression of several genes responsible for several carbohydrate cell wall and biofilm matrices components, consequently leading to resistant strains. This has been demonstrated before in gene and drug studies [126]. The variability in the susceptibility results may be related to the different antifungal drugs tested in those works.

Using a different approach, Mantri et al. [149] evaluated *Candida* sp. colonization in dentures with a silicone soft liner in diabetic and non-diabetic patients, assessing the antifungal efficacy of chlorhexidine gluconate [149]. The results showed normal oral flora in diabetics and non-diabetics and no difference between groups. They also showed a significant reduction of the colonization after cleaning the dentures with 4% chlorhexidine gluconate. This suggests that this drug has a good antiseptic effect on *Candida* sp. by killing it and preventing new adhesion. In 2015, Indira et al. [167] conducted a study that compared the common opportunistic infections (OIs) between 37 people living with HIV with DM (PLHIV-DM) and 37 people living with HIV without DM (PLHIV). Both of the groups were treated with anti-retroviral therapy (ART) [167]. The most common Ois included oral candidiasis (49% of PLHIV-DM and 35% of PLHIV) and *C. krusei* was the most common *Candida* sp. isolated (50%). No significant difference in the profile of Ois was found between PLHIV with and without DM.

#### 3.1.3. Periodontal Diseases

Periodontal diseases of fungal origin are relatively unusual in healthy individuals but arise more often in immunocompromised people or in cases when normal oral flora has been distressed (e.g., the use of broad-spectrum antibiotics) [168,169]. Diabetes is a stated risk factor for periodontitis, which is the sixth-leading complication of diabetes [170,171]. Alterations in host response, collagen metabolism, vascularity, gingival crevicular fluid, heredity patterns, and immunosuppressive treatment (drugs, dosage, and treatment duration) are known factors that promote periodontitis in diabetes [172,173]. The etiology and pathogenesis of periodontitis is still imprecise, but it is recognized that *Candida* sp. is part of the oral and subgingival microbiota of individuals who suffer from severe periodontal inflammation [174]. The virulence factors of *Candida* sp. simplify the colonization and the proliferation in the periodontal pockets by co-aggregating with bacteria in dental biofilms and adhering to epithelial cells [30], which are essential in the microbial colonization, thereby contributing to the evolution of oral diseases [175,176]. As much as 20% of patients with chronic periodontitis have been shown to have periodontal pockets that are colonized by several species of *Candida* sp., predominantly *C. albicans* [177,178], but *C. dubliniensis* [174], *C. glabrata*, and *C. tropicalis* have been reported too [176,179]. Furthermore, *C. albicans* strains isolated from subgingival sites of diabetic and periodontal patients showed high PL in cases of chronic periodontitis. Environmental oxygen concentration demonstrated influence on the virulence factors [100,180,181]. Sardi et al. [100], in a study using PCR experiments, demonstrated that the quantities of several *Candida* sp. were higher in diabetic patients with a chronic periodontal disease than in patients without diabetes. *C. albicans*, *C. dubliniensis*, *C. tropicalis*, and *C. glabrata* were detected in 57%, 75%, 16%, and 5% of the periodontal pockets, respectively. In non-diabetic patients, *C. albicans* and *C. dubliniensis* were present in 20% and 14%, respectively. Periodontal inflammation has been described to be worse in prediabetics when compared to healthy controls [182,183,184,185], assuming that the oxidative stress induced by chronic hyperglycemia with a reduced unstimulated whole salivary flow rate (UWSFR) in these patients may contribute to deteriorating periodontal status [133]. Thus, it has been suggested that glycemic control enhances healing and reduces periodontal inflammation in patients with DM and prediabetes [134,182,185,186,187,188,189,190,191]. As a result, some authors believe that it may reduce oral *Candida* sp. carriage in patients with prediabetes [133]. The HbA1c concentration is an important diagnostic tool for monitoring long-term glycemic control [192]. Also, in these cases, higher *Candida* sp. infection levels have also been associated with low diabetic control (HbA1c > 9), occurring less frequently in subjects with well-controlled blood sugar levels (HbA1c < 6).

#### 3.1.4. Denture Prosthetics and Candidiasis

Since the oral cavity is highly populated with several polymicrobial communities, each one occupies very precise niches that diverge in both anatomical location and as well as nutrient availability [193]. A consequence of strong commensal bacteria/yeast colonization is the inhibition of pathogenic microorganism colonization through resistance. The vital function of commensal yeast and bacteria and the harmful effects of commensal depletion through the use of broad spectrum antibiotics [194,195] are well recognized. Recent studies disclosed that commensal microorganisms not only protect the host by niche occupation, but also interact with host tissue, promoting the development of proper tissue structure and function [196,197].

Dentures represent a protective reservoir for oral microorganisms, mostly in biofilm form, favoring yeast proliferation, improving their infective potential, and protecting fungal cells against several medications [198,199,200,201,202]. Elderly edentulous denture wearers, patients with debilitating diseases, and users of acrylic prosthetics have a significant risk of virulent oral yeast infections [124]. Furthermore, elderly patients with diabetes have a 4.4 times higher estimated risk of developing oral candidiasis when compared with individuals without this disease. No statistically significant relation was determined between xerostomia, the use of prosthesis, and oral candidiasis [154], as suggested by some studies [203,204]. Sanitá et al. [199] studied the prevalence of *Candida* sp. in diabetics and non-diabetics with and without denture stomatitis (DS) and found that *C. albicans* was the predominant species isolated in the three groups (81.9%). They also detected *C. tropicalis* (15.71%) and *C. glabrata* (15.24%), as found in previous studies [10,14,15,21,24,57,136,144,173,205,206,207,208,209,210]. Interestingly, and contrary to other reports, the authors did not detect *C. dubliniensis* among any *Candida* sp. isolates. Even though these results confirm previous findings [10,14,16,21,24,26,136,144,173,207,209,210], this species has been isolated in both diabetic [12,57,206] and non-diabetic [208] patients. This discrepancy among studies may be related to problems with identification techniques, since *C. dubliniensis* and *C. albicans* have similar phenotypic characteristics. The same authors also found that the prevalence of *C. tropicalis* significantly increased, showing the highest degree of inflammation in DS, as observed in previous studies [12,16,24,136,144,173,208,209,210].

#### 3.1.5. Co-Occurrence of Dental Plaque, Periodontitis, and Gingivitis

The existence of numerous different oral diseases in a single patient is frequent in diabetics. Hammad et al. [29] studied the relationship between the tongue and subgingival plaque *Candida* sp. colonization, as well as its relationship with the quality of glycemic control in type 2 diabetics with periodontitis. The results showed that *Candida* sp. colonized 59% and 48.7% of the patients’ tongues and subgingival plaque, respectively. In this cross-sectional study, the authors concluded that poorly controlled type 2 diabetics and female patients with periodontitis showed a higher prevalence of subgingival plaque *Candida* sp. colonization than men, regardless of oral hygiene, tobacco smoking, age, or duration of DM [29]. The authors could not correlate oral candidal colonization and the amount of dental plaque, a patient’s gingival status, or oral hygiene, as in other studies [211], but noticed *Candida* sp. present in the dental plaque in the form of biofilm. Remarkably, and compared with the control group, they found that *C. albicans* cells isolated from the subgingival plaque of patients with periodontitis adhered more to epithelial cells [212], suggesting that the oxygen concentration in the periodontal pockets affects the virulence of *C. albicans* [213]. A study that evaluated the effect of *Candida* sp. and general disease- or treatment-related factors on plaque-related gingivitis severity in children and adolescents with Nephrotic syndrome (NS, a clinical condition with a proteinuria level exceeding the body’s compensating abilities) and diabetes concluded that poor hygiene control was the main cause of gingivitis. Olczak-Kowalczyk and colleagues [172] showed in their work that *Candida* sp. often occurred in healthy patients, but oral candidiasis was found only in the NS and diabetes groups (9.37% and 11.43%). Their work also showed that gingivitis occurred more frequently in patients with NS/diabetes. Moreover, gingivitis severity was most likely correlated to age, lipid disorders, and an increase in body mass and *Candida* sp. In uncompensated diabetes and in those patients using immunosuppressive treatment, it was assumed that NS would increase the plaque-related gingivitis.

#### 3.1.6. Esophagitis and Oropharyngeal Candidiasis

*Candida* sp. esophagitis and oropharyngeal are also oral complications found to moderately affect DM patients. Recently, Takahashi et al. [214] determined long-term trends in *Candida* sp. esophagitis (CE) prevalence and associated risk factors for patients with or without HIV infections. A risk analysis revealed that, among other factors, DM is associated with CE. Also, Mojazi and their colleagues [215] identified risk factors for oropharyngeal *Candida* sp. colonization in critically ill patients, with the results confirming DM as a risk factor [215]. Likewise, Owotade and colleagues [216] investigated the role of anti-retroviral (ARV) therapy and other factors related to oral candidiasis. Results demonstrated that 59.4% of the individuals were colonized by yeasts. *C. albicans* was the most common species (71%) and *C. dubliniensis* was the most frequent non-*Candida albicans Candida* species (NCAC). The probabilities of colonization were five times greater in patients with diabetes [216], confirming previous findings [16].

Oral and esophageal candidiasis sometimes leads to mucosal hyperplasia and progresses to carcinoma. There are many reports of the antibacterial effects of probiotics, but consensus about their antifungal effect has not been reached. In order to find alternative therapies, Terayama et al. [217] investigated whether probiotic (yogurt) containing *Lactobacillus gasseri* OLL2716 (LG21 yogurt) could prevent proliferative and inflammatory changes caused by *C. albicans* in a mucosal candidiasis animal model. Diabetes was induced in eight-week-old WBN/Kob rats by the intravenous administration of alloxan. The results suggested that probiotic (yogurt) containing *L. gasseri* OLL2716 can suppress squamous hyperplastic change and inflammation associated with *C. albicans* infection in the forestomach [217].

### 3.2. Vulvovaginal Candidiasis

The exact association between DM and vulvovaginal candidiasis (VVC) remains to be clarified, but some investigations propose that the general reduced immune response associated with DM is the main cause of recurrent VVC [218,219,220,221]. Additionally, diabetes type, severity, and degree of glucose control are probable risk factors associated with VVC prevalence, and it is acknowledged that the metabolic disorders that predispose to clinical vaginitis can be reduced by performing an appropriate diabetic control [222,223]. *C. albicans* is the most common species isolated, followed by *C. glabrata* in patients both with and without diabetes [219,224,225]. Studies have been reporting an increased frequency of infection by NCAC over time [226,227], especially *C. glabrata*, which is more recurrently associated with VVC in African and Asian countries [228,229,230,231]. Table 2 summarizes the metabolic disorders that can predispose one to VVC and those that can be decreased with proper diabetic control.

Several studies explored the association between VVC and DM. Gunther et al. [232] investigated the frequency of total isolation of vaginal *Candida* sp. and its different clinical profiles in women with type 2 DM compared to non-diabetic women in Brazil [232]. Vaginal yeast isolation occurred in 18.8% in the diabetic group and in 11.8% of women in the control group. The diabetic group was shown to be more symptomatic (VVC + recurrent VVC (RVVC) = 66.66%) than colonized women (33.33%), and indicated more colonization, VVC, and RVVC than the controls. Sherry et al. [233] studied the epidemiology of VVC in a cohort in order to find the causative organisms associated with VVC. The authors noticed a shifting prevalence of *Candida* sp. with *C. albicans* as the most common yeast but an increase of NCAC. A heterogeneous biofilm-forming capacity associated with lower antifungal drug sensitivity was also reported.

On the contrary, Ray et al. [234] stated that out of 11 diabetic patients, *C. glabrata* was isolated in 61.3% and *C. albicans* in 28.8% of VVC cases [234]. Other studies have shown similar results in diabetic women [221,235,236]. Results showed a persistent vaginal colonization with *C. glabrata* in estrogenized streptozotocin-induced type 1 diabetic mice, and vaginal polymorphonuclear (PMN) infiltration (constantly low) was found in a murine model study by Nash and colleagues [237]. Contrary to what happens in women and in other in vivo studies, in this case, curiously, biofilm formation was not detected, and co-infection of *C. glabrata* with *C. albicans* did not induce synergistic immunopathogenic effects [237].

In a different study, Nyirjesy and colleagues evaluated the effects of sodium glucose co-transporter 2 inhibitors (e.g., canagliflozin, dapagliflozin, sitagliptin) used for the treatment of type 2 DM. These drugs improve glycemic control by increasing urinary glucose excretion and are related to increased vaginal colonization with *Candida* sp. and in VVC adverse events in women with type 2 DM [252]. Of the nine subjects with VVC with a positive vaginal culture at the time of the adverse event, six cultures were positive for *C. albicans*, only one was positive for *C. glabrata*, and one was positive for *C. tropicalis*. These findings confirm previous suggestions that *C. glabrata* is less pathogenic than *C. albicans* and more often associated with asymptomatic colonization in VVC [261]. The investigation theorized that urinary glucose excretion and the subsequent deposition of urine on the vulvovaginal tissues with voiding are more significant factors in increasing the risk of VVC in diabetic women than overall glycemic control. In this study, women showed improved glycemic control due to the administration of canagliflozin [262,263] with a higher prevalence of *Candida* sp. colonization and symptomatic infection, which was also detected with dapaglifozin [264]. Also related to SGLT2 inhibitors, the prevalence and risk of VVC before SGLT2 inhibitors was carefully evaluated in real-world practice by Yokoyama and colleagues [265]. They reported that before starting SGLT2 inhibitors, 14.9% of the participants had positive vaginal *Candida* sp. colonization. Younger age and the presence of microangiopathy were significantly associated with the colonization. Moreover, of the 65 participants who were negative for *Candida* sp., 24 participants (36.9%) converted to a positive culture, and 18 participants (15.8%) developed symptomatic vaginitis. The authors concluded that the rates of developing positive colonization and symptomatic vaginitis after starting SGLT2 inhibitors appear to be higher in real-world practice than the rates of 31% and 5–10% in clinical trials, respectively. Risk factors of vaginal *Candida* colonization might be different before and after taking SGLT2 inhibitors [265].

The colonization of the vagina in prepubertal girls with *Candida* sp. is rare, as the low estrogen levels during childhood result in a rich anaerobic vaginal flora which inhibits *Candida* sp. Growth [266,267]. In a recent report, Atabek et al. [219] isolated *Candida* sp. in 39% of children with type 1 DM between 8–16 years in age. The subjects who had *Candida* sp. colonization and candidiasis were considered all acute. *C. albicans* was found in 50% of all cases, followed by *C. glabrata* (36.6%), *C. krusei* (3.3%), and *C. dubliniensis* (3.3%). Patients with VVC had a greater mean HbA1c when compared to those without such infections, and the authors thus suggested that patients with DM should undergo periodic screening for genital candidiasis [219]. Similarly, Sonck et al. [268] studied the anogenital yeast flora of 166 diabetic girls of less than 15 years of age with vulvitis, revealing that 55% were colonized, mostly by *C. albicans*.

Numerous studies have described the higher prevalence of asymptomatic vaginal colonization and symptomatic infection with *Candida* sp. in diabetic women, and some studies suggest pregnancy as an additional risk factor [254,255,256], although results are inconsistent [28,226].

Several studies have also shown that pregnancy and uncontrolled diabetes increase the infection risk. It is likely that reproductive hormone fluctuations during pregnancy and elevated glucose levels characteristic of diabetes provide the carbon needed for *Candida* overgrowth and infection. However, Sopian IL et al. [269] showed no relationship between diabetes and the occurrence of vaginal yeast infection in pregnant women, showing that there was no significant association between infection and age group, race, or education level [269]. In another report, Zheng et al. [254] studied the diversity of the vaginal fungal flora in healthy non-pregnant women, healthy pregnant women, women with gestational DM, and pregnant women with DM type 1 through an 18S rRNA gene clone library method [254]. Results showed that the most predominant vaginal fungal species belonged to the *Candida* and *Saccharomyces* genera. In a study of 251 women, Nowakowska et al. [270] demonstrated that the probability of vaginal mycosis was 4-fold greater in type 1 DM patients and nearly 2-fold greater in those with gestational DM when compared to healthy pregnant women. The report also highlighted the predominant role of poor glycemic control in the increased prevalence of vaginal candidiasis in pregnant women with type 1 DM [270]. In 2011, Masri et al. [271] determined the prevalence of *Candida* sp. in vaginal swabs of pregnant women from Serdang Hospital, Selangor, Malaysia, and their antifungal susceptibility. Results showed that 17.2% of the specimens were *Candida* sp., with *C. albicans* being the most common species detected (83.5%), followed by *C. glabrata* (16%) and *C. famata* (0.05%). All *C. albicans* and *C. famata* isolates were susceptible to fluconazole, whereas *C. glabrata* isolates had a dose-dependent susceptibility. The authors concluded that the first trimester, the second trimester, and DM were significant risk factors in patients for the vaginal candidiasis (*p* < 0.001). However, other studies noted that DM or impaired glucose tolerance during pregnancy was not connected with vaginal candidiasis [256]. Bassyouni et al. [242] explored the prevalence of VVC in diabetic women versus non-diabetic women and compared the ability of identified *Candida* sp. isolates to secrete PL and SAPs with the characterization of their genetic profile. The study involved 80 females with type 2 DM and 100 non-diabetics within the child-bearing period. Results revealed that VVC was significantly higher among the diabetic group (50%) versus the non-diabetic group (20%), and *C. albicans* was the predominant species in both groups (75% in non-diabetics and 50% in diabetics), followed by *C. glabrata* (20% in non-diabetics and 42.5% in diabetics). They also found that *Candida* sp. isolated from diabetics secreted higher quantities of proteinase than non-diabetics (87.7% and 65%, respectively), especially for *C. albicans* and *C. glabrata*, but non-significant associations between any of the tested proteinase or PL genes and DM were detected. These results were—by some means—in agreement with the ones from other reports [243,244] that also detected *C. parapsilosis* and *C. tropicalis* in a group of diabetic women. Kumari et al. [248] detected poor PL production in the isolated *Candida* sp. (causing vulvovaginitis), of which 81.25% were *C. parapsilosis*, 30.43% *C. albicans*, and 18.75% *C. glabrata*. Moreover, insignificant differences in the expression of *Candida* sp. *PLB1-2* genes and *SAP1–SAP8* genes between diabetic and non-diabetic women were reported by Bassyouni and colleagues [242]. Still, they concluded that the higher prevalence of VVC among diabetics could be directly correlated to increased SAPs production. The discrepancies between the results of different reports may be due to changes in growth conditions and host factors that alter the gene expression qualitatively and quantitatively [59,247], although findings suggest that the expression of hydrolytic enzymes by *Candida* sp. is a multifactorial process in patients with DM and the hyperglycemia level is thus not the only implicated factor.

Lastly, VVC is intimately related to vaginal mucosae biofilms. Mikamo and colleagues studied the involvement of *Candida*’s complement receptor (ICAM-1) in the adhesion of *C. albicans* or *C. glabrata* to the genitourinary epithelial cells in high glucose states. Their results demonstrated that, while the adhesion of *C. albicans* to human vaginal epithelial cells VK2/E6E7 significantly increased in the high glucose, human vulvovaginal epidermal cells A431 did not. ICAM-1 expression was increased in VK2/E6E7 cultured in the high glucose, but the expression level in A431 was not elevated. These data suggested that ICAM-1 is a ligand in the adhesion of *C. albicans* to the vaginal epithelial cells in an environment with high glucose concentration. Moreover, both host immune dysfunction and the adjustment in epithelial cells were considered responsible for VVC in diabetic patients [272].

### 3.3. Urinary Tract Candidiasis

Around 10% to 15% of in-hospital urinary tract infections (UTIs) are related to *Candida* sp. and the prevalence is still increasing [273]. Some predisposing factors such as DM, urinary retention, urinary stasis, renal transplantation, and hospitalization can increase the risk of candiduria. Specifically, DM has been known to cause severe complicated UTIs as a result of its various changes in the genitourinary system [274]. Since the 1980s, there has been a marked increase in opportunistic fungal infections involving the urinary tract, of which *C. albicans* is the most commonly isolated species, but NCAC sp. is now the majority in many countries worldwide [275,276]. Candiduria (presence of *Candida* sp. in urine) is an increasingly common finding in hospitalized patients [14], and subjects with DM are at a higher risk of developing fungal UTIs. Thus, reducing risk factors such as increasing glycemic control and the removal of urinary catheters can result in the remission of candiduria [273]. Physiopathology and etiology related to the occurrence of UTIs related to *Candida* sp. and DM are presented in Table 3.

According to the results of the Falahati et al. [277] study, there were significant associations between candiduria and the female gender, high fasting blood sugar and urine glucose, uncontrolled diabetes (HbA1c ≥ 8), and acidic urine pH (*p* < 0.05)**.** Causative agents were identified as *C. glabrata* (n = 19, 50%), *C. albicans* (n = 12, 31.6%), *C. krusei* (n = 4, 10.5%), *C. tropicalis* (n = 2, 5.3%), and *C. kefyr* (n = 1, 2.6%). The study concluded that when considering the high incidence rate of candiduria in diabetic patients, the control of diabetes, predisposing factors, and causal relationships between diabetes and candiduria should be highlighted [277]. In a 2018 study, Esmailzadeh et al. [276] evaluated candiduria among type 2 diabetic patients. Indeed, the results showed that the rate of candiduria was relatively high in type 2 diabetic patients and they were also suffering from a lack of proper blood glucose control. Although the frequency of NCAC sp. was not significantly higher than *C. albicans*, they obtained more from those with symptomatic candiduria [276]. In a cross-sectional study, Yismaw et al. [273] determined the fungal causative agents of UTIs in asymptomatic and symptomatic diabetic patients and associated risk factors. Significant candiduria was detected in 7.5% and 17.1% of asymptomatic and symptomatic type 2 diabetic patients, respectively. Among the isolated *Candida* sp., 84.2% was observed in the asymptomatic diabetic patients and the remaining 15.8% in symptomatic patients. Rizzi and Trevisan studied the prevalence and significance of UTIs and genital infections (GI) in diabetes and the effects of sodium glucose cotransporter 2 (SGLT-2) inhibitors on these complications. Results presented that diabetic patients are at high risk of UTIs and of GIs. The authors concluded that only GIs were associated with poor glycemic control. Although patients treated with SGLT-2 inhibitors have an increased 3–5 fold risk of GIs, proper medical education may reduce this risk [278].

Diabetes mellitus, indwelling bladder catheter, sex (female), and the use of antibacterial agents have been found as the risk factors identified for both *C. glabrata* and *C. albicans* candiduria [275], as previously reported [27,279]. Emphysematous cystitis, which almost exclusively occurs in diabetic patients, is rare and is seldom the result of a fungal infection [280,281]. This condition is associated with a gas formation that may present itself as cystitis, pyelitis, or pyelonephritis. Uncontrolled DM is a major risk factor for this type of infection, as it provides a favorable microenvironment in which the gas-forming organisms can grow [282,283]. Alansari et al. [284] reported a case in which a patient with uncontrolled DM was diagnosed with emphysematous pyelitis by *C. tropicalis*, while Wang et al. [285] also reported the case of a 53-year-old male patient with fungus ball and emphysematous cystitis caused concurrently by *C. tropicalis*. The predisposing factors were DM and usage of broad-spectrum antibiotics. Garg et al. [286], in a one-year prospective single center study at Dayanand Medical College and Hospital, observed 151 diabetic and non-diabetic female patients diagnosed with UTIs. Uncontrolled diabetes was more commonly associated with severe UTIs like pyelonephritis and emphysematous pyelonephritis.

*Escherichia coli* was the most frequently isolated species in both groups, followed by *Klebsiella*, *Pseudomonas*, and *Candida* sp., and the latter was only isolated from the diabetic population. Tumbarello et al. [288] identified DM and urinary catheterization as features that are specifically associated with biofilm-forming *Candida* sp. bloodstream infections. Later, Vaidyanathan et al. [281] related a case of a 58-year-old diabetic paraplegic male with a long-term indwelling urethral catheter that developed a catheter block. Results showed an *E. coli* and *C. albicans* co-infection and HbA1c (glycosylated haemoglobin) was 111 mmol/mol, which is associated with uncontrolled DM. *C. albicans* later disseminated into the bloodstream through the damaged bladder and the urethral mucosa. Moreover, those isolates formed consistently high levels of biofilm formation in vitro and a resistance to voriconazole was also detected [281]. In another report, Suzuki et al. [287] investigated the relationship between UTIs and glucosuria, observing the effect of glucosuria induced by sodium-glucose cotransporter 2 (SGLT2) inhibitors on the progression of UTIs in mice. The results showed that in mice treated with dapagliflozin and canagliflozin (not tofogliflozin), the amount of *C. albicans* colony forming units (CFU) in kidneys increased in accordance with both treatment duration and dosage. The urine glucose concentration (UGC) significantly increased up to 12 (tofogliflozin) to 24 h (dapagliflozin and canagliflozin) after SGLT2 administration, indicating that a greater susceptibility to UTIs is associated with a persistent increase in UGC [287].

### 3.4. Systemic Candidiasis

It is recognized that DM predisposes one to systemic candidiasis for several factors [289]. Among these factors, the most important are the microvascular disease progression, the low host defense mechanisms, and the diabetic vasculopathy, which exacerbates hypoperfusion and hyperglycemia and may lead to neutrophil and lymphocyte dysfunction with impaired opsonization [11,42,290,291,292,293,294,295,296,297,298,299,300,301,302,303,304,305,306,307,308,309,310,311,312,313,314,315,316,317,318,319,320,321,322]. Catheter-associated candiduria is a common clinical finding in hospitalized patients, especially in intensive care units [295], and is intimately related to biofilms. Padawer et al. [295] studied demographic and clinical data at an Israeli hospital between 2011 and 2013 on the prevalence of *Candida* sp. in catheterized in-patients and the medical interventions provided to these patients. Their results showed that candiduria was observed in 146 catheterized in-patients out of the 1408 evaluated and was directly associated with DM. *C. albicans* was present in 69.1% of the subjects, followed by *C. parapsilosis* (9.58%), *C. krusei* (7.53%), *C. tropicalis* (6.16%), *C. glabrata* (4.79%), and other species (2.73%). DM was found to be a significant risk factor of infection by *Candida* sp. In another report, Padawer et al. [295] concluded that *Candida* sp. was the second leading pathogen causing catheter-associated urinary tract infections or asymptomatic colonization. Previously, Tambyah et al. [296], Makin and Tambyah [297], and Sievert et al. [298] found similar results.

Muskett et al. [299] performed a systematic review to identify the most prevalent risk factors, looking at published analyses, risk prediction models, and clinical decision rules for invasive fungal disease (IFD) in critically ill adult patients. The authors found studies that had a significant association of DM and IFD on both univariable and multivariable analyses. Paphitou and colleagues [300] established that patients with any combination of DM, new onset hemodialysis, use of total parenteral nutrition, or receipt of broad-spectrum antibiotics had an invasive candidiasis rate of 16.6% compared to a 5.1% rate in patients lacking these characteristics (*p* = 0.001). Also, Michalopuolos et al. [301], in a univariate regression analysis study between 1997 and 2002, confirmed that DM is a significant candidemia-associated factor and an independent candidemia predictor. *C. albicans* (70%), *C. parapsilosis* (10%), *C. glabrata* (6.7%), *C. tropicalis* (6.7%), and *C. krusei* (6.7%) were isolated in patients with candidemia. *C. albicans* was simultaneously isolated from blood (89.5%) and the central venous catheter tip. Among other factors, the authors found that DM was associated with a high 30-day mortality in candidemia. Candidemia due to *C. parapsilosis* was associated with high rates of survival [11], probably due to the fact that adherence and protein secretion do not correlate with strain pathogenicity in this species as opposed to the other *Candida* sp., as had been discussed [323]. Another retrospective study from 2007 to 2015 of candidemia in hospitalized adults was performed by Khatib et al. Most of the isolates (97.5%) were *C. albicans*. *C. glabrata* was more common in diabetics (52.9% vs. 32.0% in non-diabetics; *p* = 0.004) and in abdominal sources. The findings suggested possible species-related differences in colonization dynamics or pathogenicity [324].

Abad et al. [304] carried out a different study to investigate the susceptibilities of clinical fluconazole-resistant and fluconazole-susceptible dose-dependent to caspofungin of 207 *Candida* sp. in Iranian patients. Results showed that only 9.7% of the isolates were non-sensitive to caspofungin and that these isolates were observed in cancer, DM, and AIDS patients [304]. Wu et al. [305] investigated 238 candidemia hospitalized patients between 2009 and 2011 so as to study the incidence rates of candidemia and identify the differences in risk factors of patients with *C. albicans* and NCACs and with *C. guilliermondii* and non-*C. guilliermondii* candidemia. DM was identified as a significant risk factor in patients with candidemia due to *C. albicans* (35.2%) compared to candidemia related to NCACs (13.2%). Although *C. guilliermondii* is an uncommon cause of candidemia, even in immunocompromised hosts [306,307,308,309,310,311], it was also found to occur in a significant amount of the hospitalized patients. Over the three year period, the percentage of candidemia due to *C. albicans* decreased, while the percentage of candidemia due to *C. parapsilosis* and *C. guilliermondii* increased in more than 80% of all candidemia cases in 2011 [305].

*Candida* sp. bloodstream infections (CBSI) are the fourth leading cause of nosocomial bloodstream infections in the United States [316,318,325]. CBSIs occur in up to 10% of all bloodstream infections in hospitalized patients [313,314,315], and the mortality rate is about 40% [316,317]. Normally, this mortality is higher than in bloodstream infections involving bacteria [326,327]. Risk factors for CBSIs include critically ill patients in intensive care units, DM, immunosuppressive states, mechanical ventilation, neutropenia, recent surgical procedures, and prematurity [315,318,319]. In a study by Tumbarello and colleagues [328], it was found that DM is an independent predictor of biofilm-forming *Candida* sp. CBSIs. The use of total parenteral nutrition, hospital mortality, post-CBSI hospital length of stay (LOS), and the costs of antifungal therapy were all significantly greater among patients infected by biofilm-forming isolates when compared to those infected by non-biofilm-forming isolates. It was concluded that biofilm-forming CBSI was significantly related with a high risk of death compared to non-biofilm forming CBSI [328]. Corzo-Leon and colleagues [320] investigated the clinical and epidemiologic characteristics of patients with CBSI in two tertiary care reference medical institutions in Mexico City. Their results showed that CBSIs represented 3.8% of nosocomial bloodstream infections and *C. albicans* was the predominant species (46%), followed by *C. tropicalis* (26%). *C. glabrata* was isolated from 50% of patients with diabetes and elderly patients. Nucci et al. [321] published a paper reporting an incidence of 1.18 episodes of candidemia per 1000 admissions. *C. albicans*, *C. tropicalis*, and *C. parapsilosis* were isolated in 80% of cases and DM was also found in 11% of the total cases. Gupta et al. [322] reviewed the influence of *C. glabrata* candidemia in intensive care unit (ICU) patients between 2006 and 2010. Results showed that this species was the third most isolated, and DM was a risk factor among 50% of the total cases. Also, urine was the most common source of *C. glabrata* candidemia, while the overall mortality rate was 53.8% [322]. In another report, Wang et al. [42] observed no differences in the distribution of *Candida* sp. between elderly and young patients in China, but the resistance to fluconazole and voriconazole for NCAC in the first group was double the amount of the latter. Host-related risk factors included DM, mechanical ventilation, central vascular and urethral catheter placement, and were more common in elderly patients [42].

### 3.5. Other Candidiasis

Diabetic patients are highly predisposed to cutaneous fungal infections due to the higher blood sugar levels. These infections are frequently characterized by thick biofilms, and sometimes the use of medical devices to drain these lesions is mandatory. Foot infection (tinea pedis and toenail onychomycosis) is particularly important to diabetic individuals due to the high incidence of diabetic foot in these patients [329,330]. The most significant *Candida* sp. causing onychomycosis are *C. albicans* and *C. parapsilosis* and it is known that DM patients have a high rate of tinea pedis and onychomycosis. Thus, this infection is now considered to be a predictor of diabetic foot syndrome [331]. The predisposing factors for tinea pedis et unguium are presented in Table 4. 

Diabetic foot ulcers are a serious cause of diagnostic and therapeutic concern, and Non-*albicans Candida* spp. with potential biofilm forming abilities are emerging as a predominant cause of this problematic condition. Indeed, in a recent study, the prevalence of different *Candida* sp. was identified as *C. tropicalis* (34.6%), *C. albicans* (29.3%), *C. krusei* (16.0%), *C. parapsilosis* (10.6%), and *C. glabrata* (9.33%) [332]. In order to find the frequency of fungal infections among cutaneous lesions of diabetic patients and to investigate azole antifungal agent susceptibility of the isolates, Raiesi et al. [333] studied type 1 and type 2 DM patients with foot ulcers (38.5%) and with skin and nail lesions (61.5%). Results showed that 24.5% had fungal infections and were at a higher frequency in patients with skin and nail lesions (28%) than in foot ulcers (19.1%). *C. albicans* and *Aspergillus flavus* were the most common species isolated, and a high prevalence of fluconazole-resistant *Candida* sp., particularly in diabetic foot ulcers, was determined [333].

Diabetics with onychomycosis have a greater risk of having a diabetic foot ulcer [336,337,339,340], as confirmed by numerous studies. In Germany, Eckhard and colleagues [337] found that out of 95 patients with type 1 DM, 84.6% had a fungal infection. The most frequent *Candida* sp. found were *C. albicans*, *C. parapsilosis*, and *C. guilliermondii*, followed by *C. lipolytica*, *C. catenulate*, and *C. famata*. In another study conducted at the Umm Al-Qura University, Makkah, Saudi Arabia from June 2011 to June 2012, the antimicrobial susceptibility of the most common bacterial and fungal infections among infected diabetic patients (foot infections) was determined. All *Candida* sp. showed susceptibility to amphotericin B, econazole, fluconazole, ketoconazole, and nystatin (100% each) [340]. Cases with *Candida* sp. co-infection were also observed in patients with fungal nail infections—both cutaneous and nail infections [337]. Lugo-Somolinos et al. [334] performed a cross-sectional study in Japan and revealed that 51.3% of patients with DM had onychomycosis of the toenails. In this particular case, *C. albicans* was more prevalent in the control group (24% vs. 15% in the DM patients) and nail thickness was significantly associated with an elevated HbA1c value [334]. Gupta et al. [341] reported that there was a 2.77-fold greater risk for diabetic subjects than for healthy individuals to have toenail onychomycosis. In the same year, a previous study in Taiwan reported that 60.5% of onychomycosis was caused by dermatophytes, 31.5% by *Candida* sp., and 8% by non-dermatophyte molds [342]. A total of 20 patients with onychomycosis had concomitant DM. Regarding gender, in diabetic males, the most common pathogens were dermatophytes (58.3%), while in diabetic females, *Candida* sp. was more prevalent (87.5%) [342]. However, on the contrary, Dogra and colleagues found that in Indian diabetics, yeasts were the most common isolate (48.1%), followed by dermatophytes (37%) and non-dermatophyte molds (14.8%) [336]. The authors concluded that diabetics had a 2.5 times greater probability of having onychomycosis when compared to the controls [336]. Chang et al. [331] studied 1245 Taiwanese patients with DM. Among them, 30.76% were reported to suffer from onychomycosis. In this investigation, the diagnosis of onychomycosis was limited to a general histopathologic examination (KOH stain) of the toenails. Therefore, the patients may have been affected by *Candida* sp. and by other fungi. Another study performed by Pierard et al. [335] investigated onychomycosis in 100 DM patients on chronic hemodialysis, showing that 39% of participants had a nail disease. *Candida* sp. was the second most prevalent pathogen (15%), and the authors concluded that diabetics on hemodialysis had about an 88% greater probability of acquiring onychomycosis than non-diabetics [335]. Another report by Wijesuriya and colleagues [338] described the etiological agents causing superficial fungal foot infections (SFFI) in patients with type 2 DM for one year. Their results demonstrated that 295 patients had SFFI and that, among patients with diabetes, more than 10 showed a prevalence of SFFI of 98%. *Aspergillus niger* was the most common pathogen, followed by *C. albicans*. Aging, gender, the duration of diabetes, and less-controlled glycemic levels were significantly associated with SFFIs [338].

A 2018 study explored the differential expression of toll-like receptor 2 (TLR2) and interleukin (IL)-8 secretion by keratinocytes in diabetic patients when challenged with *C. albicans*. Wang et al. [343] determined that the expression levels of both TLR2 and IL-8 increased and then decreased in the control and the diabetic groups, but in different dynamics. The observations revealed that TLR2 and IL-8 act on the keratinocytes interacting with *C. albicans*, and high glucose status can distress the function of HaCaT cells by reducing the secretion of IL-8 and TLR2. The study clearly supports the immunosuppression state that diabetic patients live in [343].

Adherence to the vascular endothelium, neutrophil chemotaxis, phagocytosis and opsonization, intracellular bactericidal activity, and cell-mediated immunity are all decreased in DM patients with hyperglycemia [344,345]. Regarding this matter, Souza et al. [346] treated diabetic rats with aminoguanidine (AMG, an inhibitor of protein glycation) and evaluated neutrophil reactive oxygen species (ROS) generation and *C. albicans* killing ability in order to evaluate the effects of hyperglycemia and the glycation of proteins on the NOX2 (phagocyte NADPH oxidase) activity of neutrophils and its implications for cellular physiology. The authors indicated that AMG increased the NOX2 response and microbicidal activity by neutrophils of the diabetic status. AMG seems to be a promising therapeutic answer for these patients [346].

In another interesting recent in vivo report, the interference of diabetic conditions in diabetic mice and the relation to the progress of *C. albicans* infection and anti-inflammatory response was evaluated. Compared to non-diabetic mice, diabetic mice indicated a significantly lower density of F4/80 and M2 macrophages, higher fungal burden, and deficiency in cytokine responses. *C. albicans* also increased tissue injury, highlighting significant deviations in diabetic animal responses to *C. albicans* infection that may be critical to the pathophysiological processes supporting cutaneous candidiasis in diabetic patients [347].

Several other pathologies related to *Candida* sp. have also been linked to a DM predisposition. Researchers often recognize the importance of DM in the development of the pathology due to immunosuppression issues [348]. The reports are summarized in Table 5.

## 4. Diabetes Mellitus In Vivo Models

Diabetes mellitus is a serious epidemic disease, and the research for new therapies is becoming critical. Thus, the correct choice of the animal models is of vital importance for the validity of the reported studies.

In DM type 1, the choices range from chemical ablation of the pancreatic β-cells to animals with spontaneously developing autoimmune diabetes. In DM type 2, the animal models can be both obese and non-obese animals with varying degrees of insulin resistance and beta cell failure [376]. The animal models (e.g., species, strain, gender, genetic) of DM type 1 and type 2 have diverse purposes, and the choice of a model depends on the purpose of the study (e.g., applied for pharmacological or genetics studies and understanding disease mechanisms) [376,377,378,379,380,381]. Table 6 summarizes the main animal models used in diabetes mellitus in vivo studies.

## 5. Conclusions

Diabetes mellitus is a severe metabolic chronic disease that is most prevalent in developed countries. The general immunocompromised state with an often-poor glucose control often leads to secondary diseases in DM individuals. The biofilm fungal infections in diabetic patients are recognized to be more complicated to treat than they are in healthy patients, especially if related to medical devices. Among the candidiasis, oral diseases are the most frequent infections that occur in DM patients, as well as VVC and, more seriously, systemic candidiasis. The reports of these cases and the results of the elected therapeutic are extremely important if we are to continue to treat these patients in the most effective manner.

## Figures and Tables

**Table 1:** Physiopathology and etiology related to the occurrence of oral candidiasis in diabetics.

	Physiopathology	Reference (s)
Uncontrolled hyperglycemia (high HbA1c) and high glucose levels in saliva	-Uncontrolled hyperglycemia may cause intensification in salivary glucose levels because in diabetics the basement membrane of the parotid salivary gland is more permeable -High glucose levels allow *Candida* sp. to multiply, even in the presence of normal bacterial flora -During hyperglycemic episodes, the chemically reversible glycosylation products with proteins in tissues and the accumulation of glycosylation products on buccal epithelial cells may sequentially increase the number of available receptors for *Candida* sp. -Glucose suppression of the killing capacity of neutrophils, emphasizing colonization (immunosuppression) -Glucose, maltose, and sucrose boost the adhesion of *Candida* to buccal epithelial cells	[15,29,31,52,134,140,141,142,143,144,145,146,147]
Lower salivary pH	-The growth of *Candida* in saliva is accompanied by a rapid decline in pH, which favors their growth and triggers the extracellular phospholipase (PL) and acid proteases, increasing the yeast adhesion to oral mucosal surfaces	[31,128,148]
Tissue response to injury is diminished	-Diabetes mellitus (DM) is known to diminish the host resistance and modify the tissue response to injury. This can result in severe colonization, even in the absence of any clinically evident oral candidiasis and possibly with further dissemination via the blood.	[130,149]
Oral epithelium	-It is most probable that the host oral epithelium of patients with diabetes favors the adhesion of colonization and subsequent infection.	[150,151]
Poor oral hygiene	The lack of control of the oral environment, especially concerning the prevention of dental caries (coronary, root, and periodontal), leads to a higher rate of oral candidiasis, especially in DM older patients	[152,153,154]
Aging	Diabetic women, orally colonized with *Candida* sp. have higher oral glucose levels than diabetics without oral *Candida* sp.	[134]
Gender		
Prostheses	Inadequate use of prostheses, together with inadequate hygienization, favours the growth of *Candida* sp.	[155,156,157]
Drugs	Xerostomia (abnomal lack if saliva): *Candida* sp. stagnation and growth on oral tissues	[30,109,110,111,136,158,159]

**Table 2:** Physiopathology and etiology related to the occurrence of vulvovaginitis in diabetics.

Condition	Physiopathology	Reference (s)
Uncontrolled hyperglycemia (high HbA1c) and high glucose levels in vaginal mucosae	- The increased serum glucose level is thought to lead to impaired monocyte, granulocyte, and neutrophil adherence, as well as reduced chemotaxis, phagocytosis, and pathogen killing - Diabetics secretions contain glucose, which can be used as a nutrient by yeasts - pH, nutritional substance, temperature, and adherence capacity in the vulvovaginal tissue may induce *Candida* sp. virulence. The vaginal epithelial cell receptor of fucose supports the adhesion of *Candida* sp. to vaginal epithelial cells -The rise in vaginal glucose and secretions and activities of hydrolytic enzymes [e.g., secreted aspartyl proteinases (SAPs), PL] increases the pathogenicity of *Candida* sp. -Increased levels of glycogen increase colonization and infection by *Candida* sp. by lowering the vaginal pH, facilitating the development of vulvovaginal candidiasis (VVC)	[55,56,59,219,222,224,225,231,238,239,240,241,242,243,244,245,246,247,248,249,250,251,252,253]
Pregnancy	-The increased circulation of estrogen levels and the deposition of glycogen and other substrates in the vagina leads to a 10–50% higher incidence of vaginal colonization by *Candida* sp. -Variability of constitutive defensins (e.g., lactoferrins, peptides) and lysozyme, leading to a poor innate immune response -Hyperglycemia can rise the anaerobic glycolysis in vaginal epithelial cells, increasing lactic acid and acetone production, decreasing the vaginal pH, thus enabling fungal colonization and proliferation	[241,254,255,256,257,258,259,260]
Diabetes type	- The incidence of VVC related to the type 1 DM or type 2 DM and is variable among studies	[218,221,222,223]
Aging	- The older one is, the higher VVC prevalence is	[222,223]

**Table 3:** Physiopathology and etiology related to the occurrence of urinary tract infections and systemic candidiasis in diabetics.

Condition	Physiopathology	Reference (s)
Uncontrolled hyperglycemia (high HbA1c) and high glucose levels in urinary tract (UT) mucosae or blood	- Favorable microenvironment for the gas-forming organisms, such as *Candida* sp., to grow	[282,283]
Gender	-An association between candiduria and being female	[27,273,279]
Drugs	-SGLT2 inhibitors (e.g., dapagliflozin, canagliflozin, tofogliflozin) administration leads to a greater susceptibility to urinary tract infection (UTI) -Association with a persistent increase in urine glucose concentration	[287]

**Table 4:** Physiopathology and etiology related to the occurrence of nail fungal diseases linked to *Candida* sp. in diabetics.

Condition	Physiopathology	Reference (s)
Uncontrolled hyperglycemia (high HbA1c) and high glucose levels in vaginal mucosae	-Circulatory disorders affecting the lower extremities (peripheral circulation), peripheral neuropathy, and retinopathy - Nail thickness is associated with an elevated HbA1c value - Diabetics using hemodialysis exhibit a higher probability of onychomycosis	[329,330,334,335,336,337,338]
Duration of DM	-More time leads to a higher probability of onychomycosis	[336]
Gender Aging	-Being male and being older are directly associated with onychomycosis in diabetics	[331,336]

**Table 5:** Physiopathology and etiology related to the occurrence of other diseases linked to *Candida* sp. in diabetics.

Condition	Physiopathology	*Candida* sp. Found in the Study	Reference (s)
Arterial infection	-Direct invasion or haematogenous spread of *Candida* sp. to the vessels wall (less common): a. infection of a pre-existing aneurysm (most possible from atherosclerotic) with fungi after an episode of candidemia b. contained fungal infection of the arterial wall (linked mostly to intravenous drug abuse).	*C. albicans* *C. tropicalis* *C. parapsilosis* Other *Candida* sp. (*not specified*)	[348,349,350,351,352,353,354,355,356,357]
Endophthalmitis	- Uncontrolled DM concomitant with other co-morbidities	*C. albicans*	[358]
Cholecystitis	- Uncontrolled DM concomitant with other co-morbidities	*C. famata*	[359]
Skin abscesses	- Uncontrolled DM concomitant with other co-morbidities	*C. albicans*	[360,361]
Brain abscesses	-Immunocompromised states and poorly-controlled diabetes (the role of DM as an immunosuppressive condition in this particular case is uncertain)	*C. albicans* *C. parapsilosis* *C. tropicalis* *C. guilliermondi* Other *Candida* sp. (*not specified*)	[362,363,364]
Fungal infections after liver transplantation	- Chronically high blood sugar may be a predisposing factor (among other factors)	*C. albicans*	[365]
Peritonitis	- Uncontrolled DM concomitant with other co-morbidities	*C. haemulonii* *C. glabrata* Other *Candida* sp. (*not specified*)	[366,367,368]
Penile prosthesis Infections		Other *Candida* sp. (*not specified*)	[369,370,371,372]
Balanitis		*C. albicans*	[373]
Fournier’s gangrene		*Candida* sp. (*not specified*)	[374]
Chronic obstructive pulmonary disease		*C. ciferrii*	[375]

**Table 6:** Main animal models used in diabetes mellitus studies [376].

Diabetes Mellitus	Model/Induction	Studies	Features
**Type 1**	*Chemical induction*		
	High dose streptozotocin	New formulations of insulin	Hyperglicaemia model
	Alloxan	Transplantation	Induced insulitis
	Multiple low dose streptozotocin	Treatment to prevent β-cell death	
	*Spontaneous autoimmune*		
	NOD mice	Understanding genetic mechanisms	Autoimmune β-cell destruction
	BB rats		
	LEW.1AR1/-iddm rats	Treatment to prevent β-cell death or to manipulate autoimmune processes	
	*Genetically Induced*		
	AKITA rats*	New formulations of insulin Transplantation Treatment to prevent ER stress	β-cell destruction due to ER + Insulin dependent
	*Virally induction*		
	Coxsackie B virus	Study of potencial role of viruses in DM type 1	Viral β-cell destruction
	Encephalomyocarditis virus		
	Kilham rat virus		
	*Lymphocytic choriomeningitis* virus (LCMV) under insulin promoter		
**Type 2**	*Monogenic—obese models*		
		Obese-induced hyperglycemia	
	*Lep^ob/ob^ mice*	Treatment to improve insulin resistance	
	*Lepr^db/db^ mice*	Treatment to improve β-cell resistance	
	*KDF rats*		
	*Polygenic—obese models*		
	*KK mice*	Treatment to insulin resistance	
	*OLEFT rat*	Treatment to improve β-cell function	
	*NZO mice*	Some models show diabetic complications	
	*TallyHO/Jng mice*		
	*NoncNZO10/|LtJ mice*		
	*Induced obesity*		
	*High fat feeding (mice or rats)*	Treatment to insulin resistance	
	*Nile grass rat*	Treatment to prevent diet-induced obesity	
	*Desert gerbil*	Treatment to improve β-cell function	

* can be also used as a DM type 2 model.

## References

[1] Willis A.M., Coulter W.A., Fulton C.R., Hayes J.R., Bell P.M., Lamey P.J. (1999). Oral candidal carriage and infection in insulin-treated diabetic patients. Diabet. Med. J. Br. Diabet. Assoc..

[2] Karaa A., Goldstein A. (2015). The spectrum of clinical presentation, diagnosis, and management of mitochondrial forms of diabetes. Pediatr. Diabetes.

[3] Calvet H.M., Yoshikawa T.T. (2001). Infections in diabetes. Infect. Dis. Clin. N. Am..

[4] Type 2 Diabetes: Prevention in People at High Risk|NICE Public Health Guideline 38—NICE. https://www.nice.org.uk/guidance/ph38/resources/type-2-diabetes-prevention-in-people-at-high-risk-pdf-1996304192197.

[5] Blake R., Trounce I.A. (2014). Mitochondrial dysfunction and complications associated with diabetes. Biochim. Biophys. Acta Gen. Subj..

[6] Tang X., Luo Y.-X., Chen H.-Z., Liu D.-P. (2014). Mitochondria, endothelial cell function, and vascular diseases. Front. Physiol..

[7] Martin S.D., McGee S.L. (2014). The role of mitochondria in the aetiology of insulin resistance and type 2 diabetes. Biochim. Biophys. Acta Gen. Subj..

[8] King H., Aubert R.E., Herman W.H. (1998). Global burden of diabetes, 1995-2025: Prevalence, numerical estimates, and projections. Diabetes Care.

[9] Agarwal S., Raman R., Paul P.G., Rani P.K., Uthra S., Gayathree R., McCarty C., Kumaramanickavel G., Sharma T. (2005). Sankara Nethralaya—Diabetic Retinopathy Epidemiology and Molecular Genetic Study (SN—DREAMS 1): Study Design and Research Methodology. Ophthalmic Epidemiol..

[10] De Resende M.A., de Sousa L.V.N.F., de Oliveira R.C.B.W., Koga-Ito C.Y., Lyon J.P. (2006). Prevalence and Antifungal Susceptibility of Yeasts Obtained from the Oral Cavity of Elderly Individuals. Mycopathologia.

[11] Guimarães T., Nucci M., Mendonça J.S., Martinez R., Brito L.R., Silva N., Moretti M.L., Salomão R., Colombo A.L. (2012). Epidemiology and predictors of a poor outcome in elderly patients with candidemia. Int. J. Infect. Dis..

[12] Khosravi A.R., Yarahmadi S., Baiat M., Shokri H., Pourkabireh M. (2008). Factors affecting the prevalence of yeasts in the oral cavity of patients with diabetes mellitus. J. Mycol. Médicale J. Med. Mycol..

[13] Tang H.J., Liu W.L., Lin H.L., Lai C.C. (2015). Epidemiology and prognostic factors of candidemia in elderly patients. Geriatr. Gerontol. Int..

[14] Belazi M., Velegraki A., Fleva A., Gidarakou I., Papanaum L., Baka D., Daniilidou N., Karamitsos D. (2005). Candidal overgrowth in diabetic patients: Potential predisposing factors. Mycoses.

[15] Darwazeh A.M.G., Lamey P.-J., Samaranayake L.P., Macfarlane T.W., Fisher B.M., Macrury S.M., Maccuish A.C. (1990). The relationship between colonisation, secretor status and in-vitro adhesion of Candida albicans to buccal epithelial cells from diabetics. J. Med. Microbiol..

[16] Gonçalves R.H.P., Miranda E.T., Zaia J.E., Giannini M.J.S.M. (2006). Species diversity of yeast in oral colonization of insulin-treated diabetes mellitus patients. Mycopathologia.

[17] Gudlaugsson O., Gillespie S., Lee K., Vande Berg J., Hu J., Messer S., Herwaldt L., Pfaller M., Diekema D. (2003). Attributable mortality of nosocomial candidemia, revisited. Clin. Infect. Dis..

[18] Kumar B.V., Padshetty N.S., Bai K.Y., Rao M.S. (2005). Prevalence of Candida in the oral cavity of diabetic subjects. J. Assoc. Physicians India.

[19] Davenport J.C. (1970). The oral distribution of candida in denture stomatitis. Br. Dent. J..

[20] Flier J.S., Underhill L.H., Brownlee M., Cerami A., Vlassara H. (1988). Advanced Glycosylation End Products in Tissue and the Biochemical Basis of Diabetic Complications. N. Engl. J. Med..

[21] Kadir T., Pisiriciler R., Akyüz S., Yarat A., Emekli N., Ipbüker A. (2002). Mycological and cytological examination of oral candidal carriage in diabetic patients and non-diabetic control subjects: Thorough analysis of local aetiologic and systemic factors. J. Oral Rehabil..

[22] Wilson R.M., Reeves W.G. (1986). Neutrophil phagocytosis and killing in insulin-dependent diabetes. Clin. Exp. Immunol..

[23] Duggan S., Essig F., Hünniger K., Mokhtari Z., Bauer L., Lehnert T., Brandes S., Häder A., Jacobsen I.D., Martin R. (2015). Neutrophil activation by Candida glabrata but not Candida albicans promotes fungal uptake by monocytes. Cell. Microbiol..

[24] Motta-Silva A.C., Aleva N.A., Chavasco J.K., Armond M.C., França J.P., Pereira L.J. (2010). Erythematous Oral Candidiasis in Patients with Controlled Type II Diabetes Mellitus and Complete Dentures. Mycopathologia.

[25] Calderone R.A., Fonzi W.A. (2001). Virulence factors of Candida albicans. Trends Microbiol..

[26] Pinto E., Ribeiro I.C., Ferreira N.J., Fortes C.E., Fonseca P.A., Figueiral M.H. (2008). Correlation between enzyme production, germ tube formation and susceptibility to fluconazole in Candida species isolated from patients with denture-related stomatitis and control individuals. J. Oral Pathol. Med..

[27] Dorko E., Baranová Z., Jenča A., Kizek P., Pilipčinec E., Tkáčiková L. (2005). Diabetes mellitus and candidiases. Folia Microbiol..

[28] Nowakowska D., Kurnatowska A., Stray-Pedersen B., Wilczyński J. (2004). Species distribution and influence of glycemic control on fungal infections in pregnant women with diabetes. J. Infect..

[29] Hammad M.M., Darwazeh A.M.G., Idrees M.M. (2013). The effect of glycemic control on Candida colonization of the tongue and the subgingival plaque in patients with type II diabetes and periodontitis. Oral Surg. Oral Med. Oral Pathol. Oral Radiol..

[30] Al Mubarak S., Robert A.A., Baskaradoss J.K., Al-Zoman K., Al Sohail A., Alsuwyed A., Ciancio S. (2013). The prevalence of oral Candida infections in periodontitis patients with type 2 diabetes mellitus. J. Infect. Public Health.

[31] Balan P., Castelino R.L., Fazil Areekat B.K. (2015). Candida Carriage Rate and Growth Characteristics of Saliva in Diabetes Mellitus Patients: A Case‒Control Study. J. Dent. Res. Dent. Clin. Dent. Prospect..

[32] Gürsoy S., Koçkar T., Atik S.U., Önal Z., Önal H., Adal E. (2018). Autoimmunity and intestinal colonization by Candida albicans in patients with type 1 diabetes at the time of the diagnosis. Korean J. Pediatr..

[33] Bommanavar S., Gugwad S., Malik N. (2017). Phenotypic switch: The enigmatic white-gray-opaque transition system of Candida albicans. J. Oral Maxillofac. Pathol..

[34] Vaarala O., Atkinson M.A., Neu J. (2008). The “Perfect Storm” for Type 1 Diabetes: The Complex Interplay Between Intestinal Microbiota, Gut Permeability, and Mucosal Immunity. Diabetes.

[35] Sapone A., de Magistris L., Pietzak M., Clemente M.G., Tripathi A., Cucca F., Lampis R., Kryszak D., Cartenì M., Generoso M. (2006). Zonulin upregulation is associated with increased gut permeability in subjects with type 1 diabetes and their relatives. Diabetes.

[36] Gosiewski T., Salamon D., Szopa M., Sroka A., Malecki M.T., Bulanda M. (2014). Quantitative evaluation of fungi of the genus Candida in the feces of adult patients with type 1 and 2 diabetes—A pilot study. Gut Pathog..

[37] Soyucen E., Gulcan A., Aktuglu-Zeybek A.C., Onal H., Kiykim E., Aydin A. (2014). Differences in the gut microbiota of healthy children and those with type 1 diabetes. Pediatr. Int..

[38] Kowalewska B., Zorena K., Szmigiero-Kawko M., Wąż P., Myśliwiec M. (2016). High Interleukin-12 Levels May Prevent an Increase in the Amount of Fungi in the Gastrointestinal Tract during the First Years of Diabetes Mellitus Type 1. Dis. Mark..

[39] Abelson J.A., Moore T., Bruckner D., Deville J., Nielsen K. (2005). Frequency of Fungemia in Hospitalized Pediatric Inpatients Over 11 Years at a Tertiary Care Institution. Pediatrics.

[40] Costa S.F., Marinho I., Araújo E.A., Manrique A.E., Medeiros E.A., Levin A.S. (2000). Nosocomial fungaemia: A 2-year prospective study. J. Hosp. Infect..

[41] Lopes Colombo A., Nucci M., Salomão R., Branchini M.L.M., Richtmann R., Derossi A., Wey S.B. (1999). High rate of non-albicans candidemia in Brazilian tertiary care hospitals. Diagn. Microbiol. Infect. Dis..

[42] Wang H., Liu N., Yin M., Han H., Yue J., Zhang F., Shan T., Guo H., Wu D. (2014). The epidemiology, antifungal use and risk factors of death in elderly patients with candidemia: A multicentre retrospective study. BMC Infect. Dis..

[43] Rodrigues C.F., Rodrigues M., Silva S., Henriques M. (2017). Candida glabrata Biofilms: How Far Have We Come?. J. Fungi.

[44] Silva S., Rodrigues C.F., Araújo D., Rodrigues M., Henriques M. (2017). Candida Species Biofilms’ Antifungal Resistance. J. Fungi.

[45] Pappas P.G., Kauffman C.A., Andes D.R., Clancy C.J., Marr K.A., Ostrosky-Zeichner L., Reboli A.C., Schuster M.G., Vazquez J.A., Walsh T.J. (2015). Clinical Practice Guideline for the Management of Candidiasis: 2016 Update by the Infectious Diseases Society of America. Clin. Infect. Dis..

[46] Hedayati M.T., Tavakoli M., Zakavi F., Shokohi T., Mofarrah R., Ansari S., Armaki M.T. (2018). In vitro antifungal susceptibility of Candida speciesisolated from diabetic patients. Rev. Soc. Bras. Med. Trop..

[47] Puig-Asensio M., Padilla B., Garnacho-Montero J., Zaragoza O., Aguado J.M., Zaragoza R., Montejo M., Muñoz P., Ruiz-Camps I., Cuenca-Estrella M. (2014). Epidemiology and predictive factors for early and late mortality in Candida bloodstream infections: A population-based surveillance in Spain. Clin. Infect. Dis..

[48] Meunier-Carpentier F., Kiehn T.E., Armstrong D. (1981). Fungemia in the immunocompromised host. Changing patterns, antigenemia, high mortality. Am. J. Med..

[49] Dimopoulos G., Karabinis A., Samonis G., Falagas M.E. (2007). Candidemia in immunocompromised and immunocompetent critically ill patients: A prospective comparative study. Eur. J. Clin. Microbiol. Infect. Dis..

[50] Man A., Ciurea C.N., Pasaroiu D., Savin A.-I., Toma F., Sular F., Santacroce L., Mare A. (2017). New perspectives on the nutritional factors influencing growth rate of Candida albicans in diabetics. An in vitro study. Mem. Inst. Oswaldo Cruz.

[51] Barnett J.A. (1981). The Utilization of Disaccharides and Some Other Sugars RY Yeasts. Adv. Carbohydr. Chem. Biochem..

[52] Rodrigues C.F., Henriques M. (2017). Oral mucositis caused by Candida glabrata biofilms: Failure of the concomitant use of fluconazole and ascorbic acid. Ther. Adv. Infect. Dis..

[53] Mandal S.M., Mahata D., Migliolo L., Parekh A., Addy P.S., Mandal M., Basak A. (2014). Glucose directly promotes antifungal resistance in the fungal pathogen, Candida spp.. J. Biol. Chem..

[54] Rodaki A., Bohovych I.M., Enjalbert B., Young T., Odds F.C., Gow N.A.R., Brown A.J.P. (2009). Glucose Promotes Stress Resistance in the Fungal Pathogen Candida albicans. Mol. Biol. Cell.

[55] Bramono K., Yamazaki M., Tsuboi R., Ogawa H. (2006). Comparison of proteinase, lipase and alpha-glucosidase activities from the clinical isolates of Candida species. Jpn. J. Infect. Dis..

[56] Ingham C.J., Boonstra S., Levels S., de Lange M., Meis J.F., Schneeberger P.M. (2012). Rapid Susceptibility Testing and Microcolony Analysis of Candida spp. Cultured and Imaged on Porous Aluminum Oxide. PLoS ONE.

[57] Manfredi M., McCullough M.J., Al-Karaawi Z.M., Hurel S.J., Porter S.R. (2002). The isolation, identification and molecular analysis of Candida spp. isolated from the oral cavities of patients with diabetes mellitus. Oral Microbiol. Immunol..

[58] Soysa N.S., Samaranayake L.P., Ellepola A.N.B. (2006). Diabetes mellitus as a contributory factor in oral candidosis. Diabet. Med..

[59] Tsang C.S.P., Chu F.C.S., Leung W.K., Jin L.J., Samaranayake L.P., Siu S.C. (2007). Phospholipase, proteinase and haemolytic activities of Candida albicans isolated from oral cavities of patients with type 2 diabetes mellitus. J. Med. Microbiol..

[60] Manns J.M., Mosser D.M., Buckley H.R. (1994). Production of a hemolytic factor by Candida albicans. Infect. Immun..

[61] Fatahinia M., Poormohamadi F., Mahmoudabadi A.Z. (2015). Comparative study of esterase and hemolytic activities in clinically important Candida species, isolated from oral cavity of diabetic and non-diabetic individuals. Jundishapur J. Microbiol..

[62] Shimizu M.T., Almeida N.Q., Fantinato V., Unterkircher C.S. (1996). Studies on hyaluronidase, chondroitin sulphatase, proteinase and phospholipase secreted by Candida species. Mycoses.

[63] Naglik J.R., Challacombe S.J., Hube B. (2003). Candida albicans secreted aspartyl proteinases in virulence and pathogenesis. Microbiol. Mol. Biol. Rev..

[64] Naglik J., Albrecht A., Bader O., Hube B. (2004). Candida albicans proteinases and host/pathogen interactions. Cell. Microbiol..

[65] Kaminishi H., Tanaka M., Cho T., Maeda H., Hagihara Y. (1990). Activation of the plasma kallikrein-kinin system by Candida albicans proteinase. Infect. Immun..

[66] Kaminishi H., Miyaguchi H., Tamaki T., Suenaga N., Hisamatsu M., Mihashi I., Matsumoto H., Maeda H., Hagihara Y. (1995). Degradation of humoral host defense by Candida albicans proteinase. Infect. Immun..

[67] Ghannoum M.A. (2000). Potential role of phospholipases in virulence and fungal pathogenesis. Clin. Microbiol. Rev..

[68] Moyes D.L., Wilson D., Richardson J.P., Mogavero S., Tang S.X., Wernecke J., Höfs S., Gratacap R.L., Robbins J., Runglall M. (2016). Candidalysin is a fungal peptide toxin critical for mucosal infection. Nature.

[69] Naglik J.R., König A., Hube B., Gaffen S.L. (2017). Candida albicans–epithelial interactions and induction of mucosal innate immunity. Curr. Opin. Microbiol..

[70] Costerton J.W., Lewandowski Z., Caldwell D.E., Korber D.R., Lappin-Scott H.M. (1995). Microbial Biofilms. Annu. Rev. Microbiol..

[71] Donlan R., Costerton J. (2002). Biofilms: Survival mechanisms of clinically relevant microorganisms. Clin. Microbiol. Rev..

[72] Fonseca E., Silva S., Rodrigues C.F., Alves C., Azeredo J., Henriques M. (2014). Effects of fluconazole on Candida glabrata biofilms and its relationship with ABC transporter gene expression. Biofouling.

[73] Rodrigues C.F., Silva S., Henriques M. (2014). Candida glabrata: A review of its features and resistance. Eur. J. Clin. Microbiol. Infect. Dis..

[74] Chandra J., Mukherjee P.K. (2015). Candida Biofilms: Development, Architecture, and Resistance. Microbiol. Spectr..

[75] Kojic E.M.E.M., Darouiche R.O.R.O. (2004). Candida infections of medical devices. Clin. Microbiol. Rev..

[76] Falagas M.E., Roussos N., Vardakas K.Z. (2010). Relative frequency of albicans and the various non-albicans Candida spp among candidemia isolates from inpatients in various parts of the world: A systematic review. Int. J. Infect. Dis..

[77] Seneviratne C.J., Jin L., Samaranayake L.P. (2008). Biofilm lifestyle of Candida: A mini review. Oral Dis..

[78] Douglas L.J. (2003). Candida biofilms and their role in infection. Trends Microbiol..

[79] Mermel L.A., Allon M., Bouza E., Craven D.E., Flynn P., O’Grady N.P., Raad I.I., Rijnders B.J.A., Sherertz R.J., Warren D.K. (2009). Clinical practice guidelines for the diagnosis and management of intravascular catheter-related infection: 2009 Update by the Infectious Diseases Society of America. Clin. Infect. Dis..

[80] Nucci M., Anaissie E., Betts R.F., Dupont B.F., Wu C., Buell D.N., Kovanda L., Lortholary O. (2010). Early Removal of Central Venous Catheter in Patients with Candidemia Does Not Improve Outcome: Analysis of 842 Patients from 2 Randomized Clinical Trials. Clin. Infect. Dis..

[81] Chandra J., Kuhn D., Mukherjee P., Hoyer L., McCormick T., Ghannoum M. (2001). Biofilm formation by the fungal pathogen Candida albicans: Development, architecture, and drug resistance. J. Bacteriol..

[82] Ramage G., Saville S.P., Thomas D.P., López-Ribot J.L. (2005). Candida biofilms: An update. Eukaryot. Cell.

[83] Andes D., Nett J., Oschel P., Albrecht R., Marchillo K., Pitula A. (2004). Development and characterization of an in vivo central venous catheter Candida albicans biofilm model. Infect. Immun..

[84] Mukherjee P.K., Chandra J. (2004). Candida biofilm resistance. Drug Resist. Updat..

[85] Nett J., Lepak A., Marchillo K., Andes D. (2009). Time course global gene expression analysis of an in vivo Candida biofilm. J. Infect. Dis..

[86] Pierce C., Vila T., Romo J., Montelongo-Jauregui D., Wall G., Ramasubramanian A., Lopez-Ribot J. (2017). The Candida albicans Biofilm Matrix: Composition, Structure and Function. J. Fungi.

[87] Rajendran R., Robertson D.P., Hodge P.J., Lappin D.F., Ramage G. (2010). Hydrolytic Enzyme Production is Associated with Candida Albicans Biofilm Formation from Patients with Type 1 Diabetes. Mycopathologia.

[88] Hoyer L.L., Cota E. (2016). Candida albicans agglutinin-like sequence (Als) family vignettes: A review of als protein structure and function. Front. Microbiol..

[89] Rauceo J.M., Gaur N.K., Lee K.-G., Edwards J.E., Klotz S.A., Lipke P.N. (2004). Global Cell Surface Conformational Shift Mediated by a Candida albicans Adhesin. Infect. Immun..

[90] Zakikhany K., Naglik J.R., Schmidt-Westhausen A., Holland G., Schaller M., Hube B. (2007). In vivo transcript profiling of Candida albicans identifies a gene essential for interepithelial dissemination. Cell. Microbiol..

[91] Zhao X., Oh S.-H., Cheng G., Green C.B., Nuessen J.A., Yeater K., Leng R.P., Brown A.J.P., Hoyer L.L. (2004). ALS3 and ALS8 represent a single locus that encodes a Candida albicans adhesin; functional comparisons between Als3p and Als1p. Microbiology.

[92] Zhao X., Oh S.-H., Yeater K.M., Hoyer L.L. (2005). Analysis of the Candida albicans Als2p and Als4p adhesins suggests the potential for compensatory function within the Als family. Microbiology.

[93] Zhao X., Oh S.-H., Hoyer L.L. (2007). Deletion of *ALS5*, *ALS6* or *ALS7* increases adhesion of *Candida albicans* to human vascular endothelial and buccal epithelial cells. Med. Mycol..

[94] Richardson J., Ho J., Naglik J. (2018). Candida–Epithelial Interactions. J. Fungi.

[95] De Las Peñas A., Pan S.-J., Castaño I., Alder J., Cregg R., Cormack B.P. (2003). Virulence-related surface glycoproteins in the yeast pathogen Candida glabrata are encoded in subtelomeric clusters and subject to RAP1- and SIR-dependent transcriptional silencing. Genes Dev..

[96] Castano I., Pan S., Zupancic M., Hennequin C., Dujon B., Cormack B. (2005). Telomere length control and transcriptional regulation of subtelomeric adhesins in Candida glabrata. Mol. Microbiol..

[97] Silva-Dias A., Miranda I.M., Branco J., Monteiro-Soares M., Pina-Vaz C., Rodrigues A.G. (2015). Adhesion, biofilm formation, cell surface hydrophobicity, and antifungal planktonic susceptibility: Relationship among Candida spp.. Front. Microbiol..

[98] De Groot P.W.J., Kraneveld E.A., Yin Q.Y., Dekker H.L., Gross U., Crielaard W., de Koster C.G., Bader O., Klis F.M., Weig M. (2008). The cell wall of the human pathogen Candida glabrata: Differential incorporation of novel adhesin-like wall proteins. Eukaryot. Cell.

[99] Ogasawara A., Odahara K., Toume M., Watanabe T., Mikami T., Matsumoto T. (2006). Change in the respiration system of Candida albicans in the lag and log growth phase. Biol. Pharm. Bull..

[100] Sardi J.C.O., Duque C., Höfling J.F., Gonçalves R.B. (2012). Genetic and phenotypic evaluation of Candida albicans strains isolated from subgingival biofilm of diabetic patients with chronic periodontitis. Med. Mycol..

[101] Calderone R.A., Braun P.C. (1991). Adherence and receptor relationships of Candida albicans. Microbiol. Rev..

[102] Silva T.M., Glee P.M., Hazen K.C. (1995). Influence of cell surface hydrophobicity on attachment of Candida albicans to extracellular matrix proteins. J. Med. Vet. Mycol..

[103] Rodrigues A.G., Mårdh P.A., Pina-Vaz C., Martinez-de-Oliveira J., Fonseca A.F. (1999). Germ tube formation changes surface hydrophobicity of Candida cells. Infect. Dis. Obstet. Gynecol..

[104] Akpan A., Morgan R. (2002). Oral candidiasis. Postgrad. Med. J..

[105] Neville B.W., Damm D.D., Allen C.M., Chi A.C. (2011). Oral and maxillofacial pathology. In Fungal and Protozoal Diseases.

[106] Regezi J.A., Sciubba J.J., Jordan R.C.K. (2008). Oral pathology clinical pathologic correlations. White Lesions.

[107] Lott T.J., Holloway B.P., Logan D.A., Fundyga R., Arnold J. (1999). Towards understanding the evolution of the human commensal yeast Candida albicans. Microbiology.

[108] Van der Meer J.W.M., van de Veerdonk F.L., Joosten L.A.B., Kullberg B.-J., Netea M.G. (2010). Severe Candida spp. infections: New insights into natural immunity. Int. J. Antimicrob. Agents.

[109] Javed F., Klingspor L., Sundin U., Altamash M., Klinge B., Engström P.-E. (2009). Periodontal conditions, oral Candida albicans and salivary proteins in type 2 diabetic subjects with emphasis on gender. BMC Oral Health.

[110] Lamey P.J., Darwaza A., Fisher B.M., Samaranayake L.P., Macfarlane T.W., Frier B.M. (1988). Secretor status, candidal carriage and candidal infection in patients with diabetes mellitus. J. Oral Pathol..

[111] Mulu A., Kassu A., Anagaw B., Moges B., Gelaw A., Alemayehu M., Belyhun Y., Biadglegne F., Hurissa Z., Moges F. (2013). Frequent detection of ‘azole’ resistant Candida species among late presenting AIDS patients in northwest Ethiopia. BMC Infect. Dis..

[112] Goregen M., Miloglu O., Buyukkurt M.C., Caglayan F., Aktas A.E. (2011). Median rhomboid glossitis: A clinical and microbiological study. Eur. J. Dent..

[113] Arendorf T.M., Walker D.M. (1984). Tobacco smoking and denture wearing as local aetiological factors in median rhomboid glossitis. Int. J. Oral Surg..

[114] Flaitz C.M., Nichols C.M., Hicks M.J. (1995). An overview of the oral manifestations of AIDS-related Kaposi’s sarcoma. Compend. Contin. Educ. Dent..

[115] Sanitá P.V., Zago C.E., Pavarina A.C., Jorge J.H., Machado A.L., Vergani C.E. (2014). Enzymatic activity profile of a Brazilian culture collection of Candida albicans isolated from diabetics and non-diabetics with oral candidiasis. Mycoses.

[116] Samaranayake L.P., Raeside J.M., MacFarlane T.W. (1984). Factors affecting the phospholipase activity of Candida species in vitro. Sabouraudia.

[117] Lyon J.P., de Resende M.A. (2006). Correlation between adhesion, enzyme production, and susceptibility to fluconazole in Candida albicans obtained from denture wearers. Oral Surg. Oral Med. Oral Pathol. Oral Radiol. Endodontol..

[118] Arslan S., Koç A.N., Şekerci A.E., Tanriverdi F., Sav H., Aydemir G., Diri H. (2016). Genotypes and virulence factors of Candida species isolated from oralcavities of patients with type 2 diabetes mellitus. Turkish J. Med. Sci..

[119] Koga-Ito C.Y., Lyon J.P., Vidotto V., de Resende M.A. (2006). Virulence Factors and Antifungal Susceptibility of Candida albicans Isolates from Oral Candidosis Patients and Control Individuals. Mycopathologia.

[120] D’Eça Júnior A., Silva A.F., Rosa F.C., Monteiro S.G., de Maria Silva Figueiredo P., de Andrade Monteiro C. (2011). In vitro differential activity of phospholipases and acid proteinases of clinical isolates of Candida. Rev. Soc. Bras. Med. Trop..

[121] Manfredi M., McCullough M.J., Al-Karaawi Z.M., Vescovi P., Porter S.R. (2006). In vitro evaluation of virulence attributes of Candida spp. isolated from patients affected by diabetes mellitus. Oral Microbiol. Immunol..

[122] De Menezes Thiele M.C., de Paula E Carvalho A., Gursky L.C., Rosa R.T., Samaranayake L.P., Rosa E.A.R. (2008). The role of candidal histolytic enzymes on denture-induced stomatitis in patients living in retirement homes. Gerodontology.

[123] Negri M., Martins M., Henriques M., Svidzinski T.I.E., Azeredo J., Oliveira R. (2010). Examination of Potential Virulence Factors of Candida tropicalis Clinical Isolates From Hospitalized Patients. Mycopathologia.

[124] Sánchez-Vargas L.O., Estrada-Barraza D., Pozos-Guillen A.J., Rivas-Caceres R. (2013). Biofilm formation by oral clinical isolates of Candida species. Arch. Oral Biol..

[125] Rodrigues C.F., Rodrigues M., Henriques M. (2018). Susceptibility of Candida glabrata biofilms to echinocandins: Alterations in the matrix composition. Biofouling.

[126] Rodrigues C.F., Henriques M. (2018). Portrait of Matrix Gene Expression in Candida glabrata Biofilms with Stress Induced by Different Drugs. Genes.

[127] Walker L.A., Gow N.A.R., Munro C.A. (2013). Elevated chitin content reduces the susceptibility of Candida species to caspofungin. Antimicrob. Agents Chemother..

[128] Samaranayake L.P., Hughes A., Weetman D.A., MacFarlane T.W. (1986). Growth and acid production of Candida species in human saliva supplemented with glucose. J. Oral Pathol..

[129] Samaranayake L.P., MacFarlane T.W. (1980). An in-vitro study of the adherence of Candida albicans to acrylic surfaces. Arch. Oral Biol..

[130] Pallavan B., Ramesh V., Dhanasekaran B.P., Oza N., Indu S., Govindarajan V. (2014). Comparison and correlation of candidal colonization in diabetic patients and normal individuals. J. Diabetes Metab. Disord..

[131] Zomorodian K., Kavoosi F., Pishdad G.R., Mehriar P., Ebrahimi H., Bandegani A., Pakshir K. (2016). Prevalence of oral Candida colonization in patients with diabetes mellitus. J. Mycol. Med..

[132] Buysschaert M., Medina J.L., Bergman M., Shah A., Lonier J. (2015). Prediabetes and associated disorders. Endocrine.

[133] Javed F., Ahmed H.B., Mehmood A., Saeed A., Al-Hezaimi K., Samaranayake L.P. (2014). Association between glycemic status and oral Candida carriage in patients with prediabetes. Oral Surg. Oral Med. Oral Pathol. Oral Radiol..

[134] Darwazeh A.M., MacFarlane T.W., McCuish A., Lamey P.J. (1991). Mixed salivary glucose levels and candidal carriage in patients with diabetes mellitus. J. Oral Pathol. Med..

[135] Yar Ahmadi S., Khosravi A., Larijani B., Baiat M., Mahmoudi M., Baradar Jalili R. (2002). Assessment of the fungal flora and the prevalence of fungal infections in the mouth of diabetics. Iran. J. Endocrinol. Metab..

[136] Fisher B.M., Lamey P.J., Samaranayake L.P., MacFarlane T.W., Frier B.M. (1987). Carriage of Candida species in the oral cavity in diabetic patients: Relationship to glycaemic control. J. Oral Pathol..

[137] Sahin I., Oksuz S., Sencan I., Gulcan A., Karabay O., Gulcan E., Yildiz O. (2005). Prevalance and risk factors for yeast colonization in adult diabetic patients. Ethiop. Med. J..

[138] Kjellman O. (1970). Secretion rate and buffering action of whole mixed saliva in subjects with insulin-treated diabetes mellitus. Odontol. Revy.

[139] Costa A.L., Silva B.M.A., Soares R., Mota D., Alves V., Mirante A., Ramos J.C., Maló de Abreu J., Santos-Rosa M., Caramelo F. (2016). Type 1 diabetes in children is not a predisposing factor for oral yeast colonization. Med. Mycol..

[140] Reinhart H., Muller G., Sobel J.D. (1985). Specificity and mechanism of in vitro adherence of Candida albicans. Ann. Clin. Lab. Sci..

[141] Samaranayake L.P., Macfarlane T.W. (1982). The Effect of Dietary Carbohydrates on the In-vitro Adhesion of Candida Albicans to Epithelial Cells. J. Med. Microbiol..

[142] Naik R., Ahmed Mujib B.R., Raaju U.R., Telagi N. (2014). Assesing oral candidal carriage with mixed salivary glucose levels as non-invasive diagnostic tool in type-2 Diabetics of Davangere, Karnataka, India. J. Clin. Diagn. Res..

[143] Sashikumar R., Kannan R. (2010). Salivary glucose levels and oral candidal carriage in type II diabetics. Oral Surg. Oral Med. Oral Pathol. Oral Radiol. Endodontol..

[144] Geerlings S.E., Hoepelman A.I. (1999). Immune dysfunction in patients with diabetes mellitus (DM). FEMS Immunol. Med. Microbiol..

[145] Ferguson D., deBurgh Norman J., McGurk M. (1995). The physiology and biology of saliva. Color Atlas and Text of Salivary Gland: Disease, Disorders and Surgery.

[146] Panchbhai A.S., Degwekar S.S., Bhowte R.R. (2010). Estimation of salivary glucose, salivary amylase, salivary total protein and salivary flow rate in diabetics in India. J. Oral Sci..

[147] Dorocka-Bobkowska B., Budtz-Jörgensen E., Włoch S. (1996). Non-insulin-dependent diabetes mellitus as a risk factor for denture stomatitis. J. Oral Pathol. Med..

[148] Samaranayake L.P., MacFarlane T.W. (1982). Factors affecting the in-vitro adherence of the fungal oral pathogen Candida albicans to epithelial cells of human origin. Arch. Oral Biol..

[149] Mantri S.P.S.S.P., Parkhedkar R.D., Mantri S.P.S.S.P. (2013). Candida colonisation and the efficacy of chlorhexidine gluconate on soft silicone-lined dentures of diabetic and non-diabetic patients. Gerodontology.

[150] Knight L., Fletcher J. (1971). Growth of Candida albicans in saliva: Stimulation by glucose associated with antibiotics, corticosteroids, and diabetes mellitus. J. Infect. Dis..

[151] Malic S., Hill K.E., Ralphs J.R., Hayes A., Thomas D.W., Potts A.J., Williams D.W. (2007). Characterization of Candida albicans infection of an in vitro oral epithelial model using confocal laser scanning microscopy. Oral Microbiol. Immunol..

[152] Wang J., Ohshima T., Yasunari U., Namikoshi S., Yoshihara A., Miyazaki H., Maeda N. (2006). The carriage of Candida species on the dorsal surface of the tongue: The correlation with the dental, periodontal and prosthetic status in elderly subjects. Gerodontology.

[153] Maria Beatriz Ribeiro Cardoso, Eliana Campêlo Lago (2010). Oral Changes in Elderly from an Association Center. Rev. Para. Med. V.

[154] Bianchi C.M., Bianchi H.A., Tadano T., Depaula C.R., Hoffmann-Santos H.D., Leite D.P., Hahn R.C. (2016). Factors related to oral candidiasis in elderly users and non-users of removable dental prostheses. Rev. Inst. Med. Trop. Sao Paulo.

[155] Prado Leite D., Rabello Piva M., Ricardo Saquete Martins-Filho P. (2015). Identification of Candida species in patients with denture stomatitis and evaluation of susceptibility to miconazole and photodynamic therapy. Rev. Odontol. UNESP.

[156] Da Silva Santos J., Batista S.A., Silva A., Ferreira M.F., Agostini M., Torres S.R. (2014). Oral candidiasis in patients admitted to ICU. Rev. Bras. Odontol..

[157] Scalercio M., Valente T., Israel M.S., Ramos M.E. (2007). Denture stomatitis associated with candidiasis: Diagnosis and treatment. RGO.

[158] Khovidhunkit S.P., Suwantuntula T., Thaweboon S., Mitrirattanakul S., Chomkhakhai U., Khovidhunkit W. (2009). Xerostomia, hyposalivation, and oral microbiota in type 2 diabetic patients: A preliminary study. J. Med. Assoc. Thail..

[159] Sudbery P., Gow N., Berman J. (2004). The distinct morphogenic states of Candida albicans. Trends Microbiol..

[160] Aitken-Saavedra J., Lund R.G., González J., Huenchunao R., Perez-Vallespir I., Morales-Bozo I., Urzúa B., Tarquinio S.C., Maturana-Ramírez A., Martos J. (2018). Diversity, frequency and antifungal resistance of *Candida* species in patients with type 2 diabetes mellitus. Acta Odontol. Scand..

[161] Lydia Rajakumari M., Saravana Kumari P. (2016). Prevalence of Candida species in the buccal cavity of diabetic and non-diabetic individuals in and around Pondicherry. J. Mycol. Med..

[162] Premkumar J., Ramani P., Chandrasekar T., Natesan A., Premkumar P. (2014). Detection of species diversity in oral candida colonization and anti-fungal susceptibility among non-oral habit adult diabetic patients. J. Nat. Sci. Biol. Med..

[163] Bremenkamp R.M., Caris A.R., Jorge A.O.C., Back-Brito G.N., Mota A.J., Balducci I., Brighenti F.L., Koga-Ito C.Y. (2011). Prevalence and antifungal resistance profile of Candida spp. oral isolates from patients with type 1 and 2 diabetes mellitus. Arch. Oral Biol..

[164] Manfredi M., McCullough M.J., Polonelli L., Conti S., Al-Karaawi Z.M., Vescovi P., Porter S.R. (2006). In vitro antifungal susceptibility to six antifungal agents of 229 Candida isolates from patients with diabetes mellitus. Oral Microbiol. Immunol..

[165] Sanitá P.V., De Oliveira Mima E.G., Pavarina A.C., Jorge J.H., Machado A.L., Vergani C.E. (2013). Susceptibility profile of a Brazilian yeast stock collection of Candida species isolated from subjects with Candida-associated denture stomatitis with or without diabetes. Oral Surg. Oral Med. Oral Pathol. Oral Radiol..

[166] Al-Attas S., Amro S. (2010). Candidal colonization, strain diversity, and antifungal susceptibility among adult diabetic patients. Ann. Saudi Med..

[167] Indira P., Kumar P.M., Shalini S., Vaman K. (2015). Opportunistic infections among People Living with HIV (PLHIV) with Diabetes Mellitus (DM) attending a tertiary care hospital in coastal city of South India. PLoS ONE.

[168] Scully C., Monteil R., Sposto M.R. (1998). Infectious and tropical diseases affecting the human mouth. Periodontology 2000.

[169] Stanford T.W., Rivera-Hidalgo F. (1999). Oral mucosal lesions caused by infective microorganisms. II. Fungi and parasites. Periodontology 2000.

[170] Löe H. (1993). Periodontal disease. The sixth complication of diabetes mellitus. Diabetes Care.

[171] Cullinan M., Ford P., Seymour G. (2009). Periodontal disease and systemic health: Current status. Aust. Dent. J..

[172] Olczak-Kowalczyk D., Pyrżak B., Dąbkowska M., Pańczyk-Tomaszewska M., Miszkurka G., Rogozińska I., Swoboda-Kopeć E., Gozdowski D., Kalińska A., Piróg A. (2015). Candida spp. and gingivitis in children with nephrotic syndrome or type 1 diabetes. BMC Oral Health.

[173] Lotfi-Kamran M.H., Jafari A.A., Falah-Tafti A., Tavakoli E., Falahzadeh M.H. (2009). Candida Colonization on the Denture of Diabetic and Non-diabetic Patients. Dent. Res. J..

[174] Urzúa B., Hermosilla G., Gamonal J., Morales-Bozo I., Canals M., Barahona S., Cóccola C., Cifuentes V. (2008). Yeast diversity in the oral microbiota of subjects with periodontitis: *Candida albicans* and *Candida dubliniensis* colonize the periodontal pockets. Med. Mycol..

[175] Sardi J., Duque C., Mariano F., Peixoto I., Hofling J., Gonçalves R.B. (2010). Candida spp. in periodontal disease: A brief review. J. Oral Sci..

[176] Järvensivu A., Hietanen J., Rautemaa R., Sorsa T., Richardson M. (2004). Candida yeasts in chronic periodontitis tissues and subgingival microbial biofilms in vivo. Oral Dis..

[177] Slots J., Rams T.E., Listgarten M.A. (1988). Yeasts, enteric rods and pseudomonads in the subgingival flora of severe adult periodontitis. Oral Microbiol. Immunol..

[178] Reynaud A.H., Nygaard-Østby B., Bøygard G.K., Eribe E.R., Olsen I., Gjermo P. (2001). Yeasts in periodontal pockets. J. Clin. Periodontol..

[179] Ergun S., Cekici A., Topcuoglu N., Migliari D.-A., Külekçi G., Tanyeri H., Isik G. (2010). Oral status and Candida colonization in patients with Sjögren’s Syndrome. Med. Oral Patol. Oral Cir. Bucal.

[180] Sardi J.C.O., Duque C., Camargo G.A.C.G., Hofling J.F., Gonçalves R.B. (2011). Periodontal conditions and prevalence of putative periodontopathogens and Candida spp. in insulin-dependent type 2 diabetic and non-diabetic patients with chronic periodontitis—A pilot study. Arch. Oral Biol..

[181] Barros L.M., Boriollo M.F.G., Alves A.C.B.A., Klein M.I., Gonçalves R.B., Höfling J.F. (2008). Genetic diversity and exoenzyme activities of Candida albicans and Candida dubliniensis isolated from the oral cavity of Brazilian periodontal patients. Arch. Oral Biol..

[182] Javed F., Thafeed AlGhamdi A.S., Mikami T., Mehmood A., Ahmed H.B., Samaranayake L.P., Tenenbaum H.C. (2014). Effect of Glycemic Control on Self-Perceived Oral Health, Periodontal Parameters, and Alveolar Bone Loss Among Patients With Prediabetes. J. Periodontol..

[183] Javed F., Samaranayake L.P., Al-Askar M., Al-Hezaimi K. (2013). Periodontal Disease in Habitual Cigarette Smokers and Nonsmokers With and Without Prediabetes. Am. J. Med. Sci..

[184] Javed F., Tenenbaum H.C., Nogueira-Filho G., Nooh N., O’Bello Correa F., Warnakulasuriya S., Dasanayake A.P., Al-Hezaimi K. (2013). Periodontal Inflammatory Conditions Among Gutka Chewers and Non-chewers With and Without Prediabetes. J. Periodontol..

[185] Javed F., Al-Askar M., Al-Rasheed A., Al-Hezaimi K., Babay N., Galindo-Moreno P. (2012). Comparison of Self-Perceived Oral Health, Periodontal Inflammatory Conditions and Socioeconomic Status in Individuals With and Without Prediabetes. Am. J. Med. Sci..

[186] Javed F., Näsström K., Benchimol D., Altamash M., Klinge B., Engström P.-E. (2007). Comparison of Periodontal and Socioeconomic Status Between Subjects With Type 2 Diabetes Mellitus and Non-Diabetic Controls. J. Periodontol..

[187] Javed F., Romanos G.E. (2009). Impact of Diabetes Mellitus and Glycemic Control on the Osseointegration of Dental Implants: A Systematic Literature Review. J. Periodontol..

[188] Bader M.S., Hinthorn D., Lai S.M., Ellerbeck E.F. (2005). Hyperglycaemia and mortality of diabetic patients with candidaemia. Diabet. Med..

[189] Oztürkcan S., Oztürkcan S., Topçu S., Akinci S., Bakici M.Z., Yalçin N. (1993). Incidence of oral candidiasis in diabetic patients. Mikrobiyol. Bülteni.

[190] Rodero L., Davel G., Soria M., Vivot W., Córdoba S., Canteros C.E., Saporiti A., EMIFN (2005). [Multicenter study of fungemia due to yeasts in Argentina]. Rev. Argent. Microbiol..

[191] Saes Busato I.M., Bittencourt M.S., Machado M.Â.N., Grégio A.M.T., Azevedo-Alanis L.R. (2010). Association between metabolic control and oral health in adolescents with type 1 diabetes mellitus. Oral Surg. Oral Med. Oral Pathol. Oral Radiol. Endodontol..

[192] Weykamp C. (2013). HbA1c: A review of analytical and clinical aspects. Ann. Lab. Med..

[193] Marsh P.D. (1994). Microbial Ecology of Dental Plaque and its Significance in Health and Disease. Adv. Dent. Res..

[194] Brook I. (1999). Bacterial Interference. Crit. Rev. Microbiol..

[195] He X., McLean J.S., Guo L., Lux R., Shi W. (2014). The social structure of microbial community involved in colonization resistance. ISME J..

[196] Roberts F.A., Darveau R.P. (2015). Microbial protection and virulence in periodontal tissue as a function of polymicrobial communities: Symbiosis and dysbiosis. Periodontology 2000.

[197] Ley R.E., Hamady M., Lozupone C., Turnbaugh P.J., Ramey R.R., Bircher J.S., Schlegel M.L., Tucker T.A., Schrenzel M.D., Knight R. (2008). Evolution of Mammals and Their Gut Microbes. Science.

[198] Mima E.G.G., Vergani C.E.E., Machado A.L.L., Massucato E.M.S.M.S., Colombo A.L.L., Bagnato V.S.S., Pavarina A.C.C. (2012). Comparison of Photodynamic Therapy versus conventional antifungal therapy for the treatment of denture stomatitis: A randomized clinical trial. Clin. Microbiol. Infect..

[199] Sanit P.V., Pavarina A.C., Giampaolo E.T., Silva M.M., De Oliveira Mima E.G., Ribeiro D.G., Vergani C.E. (2011). Candida spp. prevalence in well controlled type 2 diabetic patients with denture stomatitis. Oral Surg. Oral Med. Oral Pathol. Oral Radiol. Endodontol..

[200] Sanita P.V., Machado A.L., Pavarina A.C., Massucato E.M.S., Colombo A.L., Vergani C.E. (2012). Microwave denture disinfection versus nystatin in treating patients with well-controlled type 2 diabetes and denture stomatitis: A randomized clinical trial. Int. J. Prosthodont..

[201] Silva M.M., Mima E.G., Colombo A.L., Sanitá P.V., Jorge J.H., Massucato E.M.S., Vergani C.E. (2012). Comparison of denture microwave disinfection and conventional antifungal therapy in the treatment of denture stomatitis: A randomized clinical study. Oral Surg. Oral Med. Oral Pathol. Oral Radiol..

[202] Melo A.S., Bizerra F.C., Freymüller E., Arthington-Skaggs B.A., Colombo A.L. (2011). Biofilm production and evaluation of antifungal susceptibility amongst clinical *Candida* spp. isolates, including strains of the *Candida parapsilosis* complex. Med. Mycol..

[203] Iacopino A.M., Wathen W.F. (1992). Oral candidal infection and denture stomatitis: A comprehensive review. J. Am. Dent. Assoc..

[204] Artico G., Freitas R., Santos Filho A., Benard G., Romiti R., Migliari D. (2014). Prevalence of *Candida* spp., xerostomia, and hyposalivation in oral lichen planus—A controlled study. Oral Dis..

[205] Willis A.M., Coulter W.A., Sullivan D.J., Coleman D.C., Hayes J.R., Bell P.M., Lamey P.J. (2000). Isolation of C. dubliniensis from insulin-using diabetes mellitus patients. J. Oral Pathol. Med..

[206] Coco B.J., Bagg J., Cross L.J., Jose A., Cross J., Ramage G. (2008). Mixed Candida albicans and Candida glabrata populations associated with the pathogenesis of denture stomatitis. Oral Microbiol. Immunol..

[207] Marcos-Arias C., Vicente J.L., Sahand I.H., Eguia A., De-Juan A., Madariaga L., Aguirre J.M., Eraso E., Quindós G. (2009). Isolation of Candida dubliniensis in denture stomatitis. Arch. Oral Biol..

[208] Vanden Abbeele A., de Meel H., Ahariz M., Perraudin J.-P., Beyer I., Courtois P. (2008). Denture contamination by yeasts in the elderly. Gerodontology.

[209] Webb B.C., Thomas C.J., Whittle T. (2005). A 2-year study of Candida-associated denture stomatitis treatment in aged care subjects. Gerodontology.

[210] Fongsmut T., Deerochanawong C., Prachyabrued W. (1998). Intraoral candida in Thai diabetes patients. J. Med. Assoc. Thail..

[211] Darwazeh A.-G., Hammad M., Al-Jamaei A. (2010). The relationship between oral hygiene and oral colonization with *Candida* species in healthy adult subjects. Int. J. Dent. Hyg..

[212] Machado A.G., Komiyama E.Y., Dos Santos S.S.F., Jorge A.O.C., Brighenti F.L., Koga-Ito C.Y. (2011). In vitro adherence of Candida albicans isolated from patients with chronic periodontitis. J. Appl. Oral Sci..

[213] Rosa E.A.R., Rached R.N., Ignacio S.A., Rosa R.T., Jose da Silva W., Yau J.Y.Y., Samaranayake L.P. (2008). Phenotypic evaluation of the effect of anaerobiosis on some virulence attributes of Candida albicans. J. Med. Microbiol..

[214] Takahashi Y., Nagata N., Shimbo T., Nishijima T., Watanabe K., Aoki T., Sekine K., Okubo H., Watanabe K., Sakurai T. (2015). Long-term trends in esophageal candidiasis prevalence and associated risk factors with or without HIV infection: Lessons from an endoscopic study of 80,219 patients. PLoS ONE.

[215] Mojazi Amiri H., Frandah W., Colmer-Hamood J., Raj R., Nugent K. (2012). Risk factors of Candida colonization in the oropharynx of patients admitted to an intensive care unit. J. Mycol. Med..

[216] Owotade F.J., Patel M., Ralephenya T.R.M.D.R., Vergotine G. (2013). Oral candida colonization in HIV-positive women: Associated factors and changes following antiretroviral therapy. J. Med. Microbiol..

[217] Terayama Y., Matsuura T., Uchida M., Narama I., Ozaki K. (2016). Probiotic (yogurt) containing Lactobacillus gasseri OLL2716 is effective for preventing Candida albicans-induced mucosal inflammation and proliferation in the forestomach of diabetic rats. Histol. Histopathol..

[218] Malazy O.T., Shariat M., Heshmat R., Majlesi F., Alimohammadian M., Tabari N.K., Larijani B. (2007). Vulvovaginal candidiasis and its related factors in diabetic women. Taiwan J. Obstet. Gynecol..

[219] Atabek M.E., Akyürek N., Eklioglu B.S. (2013). Frequency of Vaginal Candida Colonization and Relationship between Metabolic Parameters in Children with Type 1 Diabetes Mellitus. J. Pediatr. Adolesc. Gynecol..

[220] Bohannon N.J. (1998). V Treatment of Vulvovaginal Candidiasis in Patients With Diabetes. Diabetes Care.

[221] De Leon E.M., Jacober S.J., Sobel J.D., Foxman B. (2002). Prevalence and risk factors for vaginal Candida colonization in women with type 1 and type 2 diabetes. BMC Infect. Dis..

[222] Reed B.D. (1992). Risk factors for Candida vulvovaginitis. Obstet. Gynecol. Surv..

[223] Hoeltge G. (1996). Clinical Laboratory Technical Procedure Manuals.

[224] Saporiti A.M., Gómez D., Levalle S., Galeano M., Davel G., Vivot W., Rodero L. (2001). [Vaginal candidiasis: Etiology and sensitivity profile to antifungal agents in clinical use]. Rev. Argent. Microbiol..

[225] Sobel J.D., Chaim W. (1997). Treatment of Torulopsis glabrata vaginitis: Retrospective review of boric acid therapy. Clin. Infect. Dis..

[226] Goswami R., Dadhwal V., Tejaswi S., Datta K., Paul A., Haricharan R.N., Banerjee U., Kochupillai N.P. (2000). Species-specific prevalence of vaginal candidiasis among patients with diabetes mellitus and its relation to their glycaemic status. J. Infect..

[227] Nagesha C.N., Ananthakrishna N.C. (1970). Clinical and laboratory study of monilial vaginitis. Am. J. Obstet. Gynecol..

[228] Grigoriou O., Baka S., Makrakis E., Hassiakos D., Kapparos G., Kouskouni E. (2006). Prevalence of clinical vaginal candidiasis in a university hospital and possible risk factors. Eur. J. Obstet. Gynecol. Reprod. Biol..

[229] Achkar J.M., Fries B.C. (2010). Candida infections of the genitourinary tract. Clin. Microbiol. Rev..

[230] Deorukhkar S.C., Saini S., Mathew S. (2014). Non-albicans Candida Infection: An Emerging Threat. Interdiscip. Perspect. Infect. Dis..

[231] Lattif A.A., Mukhopadhyay G., Banerjee U., Goswami R., Prasad R. (2011). Molecular typing and in vitro fluconazole susceptibility of Candida species isolated from diabetic and nondiabetic women with vulvovaginal candidiasis in India. J. Microbiol. Immunol. Infect..

[232] Gunther L.S.A., Martins H.P.R., Gimenes F., De Abreu A.L.P., Consolaro M.E.L., Svidzinski T.I.E. (2014). Prevalence of Candida albicans and non-albicans isolates from vaginal secretions: Comparative evaluation of colonization, vaginal candidiasis and recurrent vaginal candidiasis in diabetic and non-diabetic women. Sao Paulo Med. J..

[233] Sherry L., Kean R., McKloud E., O’Donnell L.E., Metcalfe R., Jones B.L., Ramage G. (2017). Biofilms Formed by Isolates from Recurrent Vulvovaginal Candidiasis Patients Are Heterogeneous and Insensitive to Fluconazole. Antimicrob. Agents Chemother..

[234] Ray D., Goswami R., Banerjee U., Dadhwal V., Goswami D., Mandal P., Sreenivas V., Kochupillai N. (2007). Prevalence of Candida glabrata and Its Response to Boric Acid Vaginal Suppositories in Comparison With Oral Fluconazole in Patients With Diabetes and Vulvovaginal Candidiasis. Diabetes Care.

[235] Peer A.K., Hoosen A.A., Seedat M.A., van den Ende J., Omar M.A. (1993). Vaginal yeast infections in diabetic women. S. Afr. Med. J..

[236] Nyirjesy P., Sobel J.D. (2013). Genital Mycotic Infections in Patients With Diabetes. Postgrad. Med..

[237] Nash E.E., Peters B.M., Lilly E.A., Noverr M.C., Fidel P.L. (2016). A Murine Model of Candida glabrata Vaginitis Shows No Evidence of an Inflammatory Immunopathogenic Response. PLoS ONE.

[238] Corrêa P.R., David P.R., Peres N.P., da Cunha K.C., de Almeida M.T.G. (2009). [Phenotypic characterization of yeasts isolated from the vaginal mucosa of adult women]. Rev. Bras. Ginecol. Obstet..

[239] Carrara M.A., Bazotte R.B., Donatti L., Svidzinski T.I.E., Consolaro M.E.L., Patussi E.V., Batista M.R. (2009). Effect of experimental diabetes on the development and maintenance of vulvovaginal candidiasis in female rats. Am. J. Obstet. Gynecol..

[240] Bassyouni R.H., Wegdan A.A., Abdelmoneim A., Said W., Aboelnaga F. (2015). Phospholipase and aspartyl proteinase activities of candida species causing vulvovaginal candidiasis in patients with type 2 diabetes mellitus. J. Microbiol. Biotechnol..

[241] Faraji R., Rahimi A., Rezvanmadani F., Hashemi M. (2012). Prevalence of vaginal candidiasis infection in diabetic women. Afr. J. Microbiol. Res..

[242] Yildirim Z., Kilic N., Kalkanci A. (2011). Fluorometric determination of acid proteinase activity in Candida albicans strains from diabetic patients with vulvovaginal candidiasis. Mycoses.

[243] Chaffin W.L. (2008). Candida albicans cell wall proteins. Microbiol. Mol. Biol. Rev..

[244] Yang Y.-L. (2003). Virulence factors of Candida species. J. Microbiol. Immunol. Infect..

[245] Samaranayake Y.H., Dassanayake R.S., Cheung B.P.K., Jayatilake J.A.M.S., Yeung K.W.S., Yau J.Y.Y., Samaranayake L.P. (2006). Differential phospholipase gene expression by *Candida albicans* in artificial media and cultured human oral epithelium. APMIS.

[246] Tilak R., Kumari V., Banerjee T., Kumar P., Pandey S. (2013). Emergence of non-albicans Candida among candidal vulvovaginitis cases and study of their potential virulence factors, from a tertiary care center, North India. Indian J. Pathol. Microbiol..

[247] Kuştimur S., El-Nahi H., Altan N. (1991). Virulence of Proteinase-Positive and Proteinase-Negative Candida Albicans to Mouse and Killing of the Yeast by Normal Human Leukocytes. Candida and Candidamycosis.

[248] Kendirci M., Koç A.N., Kurtoglu S., Keskin M., Kuyucu T. (2004). Vulvovaginal candidiasis in children and adolescents with type 1 diabetes mellitus. J. Pediatr. Endocrinol. Metab..

[249] Kelekci S., Kelekci H., Cetin M., Inan I., Tokucoglu S. (2004). Glucose tolerance in pregnant women with vaginal candidiasis. Ann. Saudi Med..

[250] Nyirjesy P., Zhao Y., Ways K., Usiskin K. (2012). Evaluation of vulvovaginal symptoms and Candida colonization in women with type 2 diabetes mellitus treated with canagliflozin, a sodium glucose co-transporter 2 inhibitor. Curr. Med. Res. Opin..

[251] Raith L., Csató M., Dobozy A. (1983). Decreased Candida albicans killing activity of granulocytes from patients with diabetes mellitus. Mykosen.

[252] Paramythiotou E., Frantzeskaki F., Flevari A., Armaganidis A., Dimopoulos G. (2014). Invasive Fungal Infections in the ICU: How to Approach, How to Treat. Molecules.

[253] Sobel J.D., Myers P.G., Kaye D., Levison M.E. (1981). Adherence of Candida albicans to human vaginal and buccal epithelial cells. J. Infect. Dis..

[254] Zheng N.N., Guo X.C., Lv W., Chen X.X., Feng G.F. (2013). Characterization of the vaginal fungal flora in pregnant diabetic women by 18S rRNA sequencing. Eur. J. Clin. Microbiol. Infect. Dis..

[255] Nowakowska D., Kurnatowska A., Stray-Pedersen B., Wilczyński J. (2004). Activity of hydrolytic enzymes in fungi isolated from diabetic pregnant women: Is there any relationship between fungal alkaline and acid phosphatase activity and glycemic control?. APMIS.

[256] Guzel A.B., Ilkit M., Burgut R., Urunsak İ.F., Ozgunen F.T. (2011). An Evaluation of Risk Factors in Pregnant Women with Candida Vaginitis and the Diagnostic Value of Simultaneous Vaginal and Rectal Sampling. Mycopathologia.

[257] Hay P., Czeizel A.E. (2007). Asymptomatic trichomonas and candida colonization and pregnancy outcome. Best Pract. Res. Clin. Obstet. Gynaecol..

[258] Spinillo A., Capuzzo E., Acciano S., De Santolo A., Zara F. (1999). Effect of antibiotic use on the prevalence of symptomatic vulvovaginal candidiasis. Am. J. Obstet. Gynecol..

[259] Cotch M.F., Hillier S.L., Gibbs R.S., Eschenbach D.A. (1998). Epidemiology and outcomes associated with moderate to heavy Candida colonization during pregnancy. Vaginal Infections and Prematurity Study Group. Am. J. Obstet. Gynecol..

[260] French W., Gad A. (1977). The frequency of Candida infections in pregnancy and their treatment with clotrimazole. Curr. Med. Res. Opin..

[261] Huang A.J., Moore E.E., Boyko E.J., Scholes D., Lin F., Vittinghoff E., Fihn S.D. (2010). Vaginal symptoms in postmenopausal women. Menopause.

[262] Rosenstock J., Polidori D., Zhao Y., Al E. Canagliflozin, an inhibitor of sodium glucose co-transporter 2, improves glycemic control, lowers body weight, and improves beta-cell function in subjects with type 2 diabetes on background metformin. Proceedings of the 46th Annual Meeting of the European Association for the Study of Diabetes.

[263] Rosenstock J., Aggarwal N., Polidori D., Zhao Y., Arbit D., Usiskin K., Capuano G., Canovatchel W. (2012). Canagliflozin DIA 2001 Study Group Dose-Ranging Effects of Canagliflozin, a Sodium-Glucose Cotransporter 2 Inhibitor, as Add-On to Metformin in Subjects With Type 2 Diabetes. Diabetes Care.

[264] Bailey C.J., Gross J.L., Pieters A., Bastien A., List J.F. (2010). Effect of dapagliflozin in patients with type 2 diabetes who have inadequate glycaemic control with metformin: A randomised, double-blind, placebo-controlled trial. Lancet.

[265] Yokoyama H., Nagao A., Watanabe S., Honjo J. (2018). Incidence and risk of vaginal candidiasis associated with sodium-glucose cotransporter 2 inhibitors in real-world practice for women with type 2 diabetes. J. Diabetes Investig..

[266] Banerjee K., Curtis E., San Lazaro C., Graham J.C. (2004). Low prevalence of genital candidiasis in children. Eur. J. Clin. Microbiol. Infect. Dis..

[267] Schaaf V.M., Perez-Stable E.J., Borchardt K. (1990). The limited value of symptoms and signs in the diagnosis of vaginal infections. Arch. Intern. Med..

[268] Sonck C.E., Somersalo O. (1963). The yeast flora of the anogenital region in diabetic girls. Arch. Dermatol..

[269] Sopian I.L., Shahabudin S., Ahmed M.A., Lung L.T.T., Sandai D. (2016). Yeast Infection and Diabetes Mellitus among Pregnant Mother in Malaysia. Malays. J. Med. Sci..

[270] Nowakowska D., Kurnatowska A., Stray-Pedersen B., Wilczynski J. (2004). Prevalence of fungi in the vagina, rectum and oral cavity in pregnant diabetic women: Relation to gestational age and symptoms. Acta Obstet. Gynecol. Scand..

[271] Masri S.N., Noor S.M., Mat Nor L.A., Osman M., Rahman M.M. (2015). Candida isolates from pregnant women and their antifungal susceptibility in a Malaysian tertiary-care hospital. Pakistan J. Med. Sci..

[272] Mikamo H., Yamagishi Y., Sugiyama H., Sadakata H., Miyazaki S., Sano T., Tomita T. (2018). High glucose-mediated overexpression of ICAM-1 in human vaginal epithelial cells increases adhesion of *Candida albicans*. J. Obstet. Gynaecol..

[273] Yismaw G., Asrat D., Woldeamanuel Y., Unakal C. (2013). Prevalence of candiduria in diabetic patients attending Gondar University Hospital, Gondar, Ethiopia. Iran. J. Kidney Dis..

[274] Mnif M.F., Kamoun M., Kacem F.H., Bouaziz Z., Charfi N., Mnif F., Ben Naceur B., Rekik N., Abid M. (2013). Complicated urinary tract infections associated with diabetes mellitus: Pathogenesis, diagnosis and management. Indian J. Endocrinol. Metab..

[275] Sobel J.D. (1997). Vaginitis. N. Engl. J. Med..

[276] Esmailzadeh A., Zarrinfar H., Fata A., Sen T. (2018). High prevalence of candiduria due to non- *albicans Candida* species among diabetic patients: A matter of concern?. J. Clin. Lab. Anal..

[277] Falahati M., Farahyar S., Akhlaghi L., Mahmoudi S., Sabzian K., Yarahmadi M., Aslani R. (2016). Characterization and identification of candiduria due to Candida species in diabetic patients. Curr. Med. Mycol..

[278] Rizzi M., Trevisan R. (2016). Genitourinary infections in diabetic patients in the new era of diabetes therapy with sodium-glucose cotransporter-2 inhibitors. Nutr. Metab. Cardiovasc. Dis..

[279] Jarvis W.R. (1995). Epidemiology of nosocomial fungal infections, with emphasis on Candida species. Clin. Infect. Dis..

[280] Bartkowski D.P., Lanesky J.R. (1988). Emphysematous prostatitis and cystitis secondary to Candida albicans. J. Urol..

[281] Vaidyanathan S., Soni B., Hughes P., Ramage G., Sherry L., Singh G., Mansour P. (2013). Candida albicans Fungaemia following Traumatic Urethral Catheterisation in a Paraplegic Patient with Diabetes Mellitus and Candiduria Treated by Caspofungin. Case Rep. Infect. Dis..

[282] Huang J.J., Tseng C.C. (2000). Emphysematous pyelonephritis: Clinicoradiological classification, management, prognosis, and pathogenesis. Arch. Intern. Med..

[283] Grupper M., Kravtsov A., Potasman I. (2007). Emphysematous Cystitis. Medicine.

[284] Alansari A., Borras M.D., Boma N. (2015). “I have chicken fat in my urine!” A case of Candida tropicalis induced emphysematous pyelitis. Med. Mycol. Case Rep..

[285] Wang L., Ji X., Sun G., Qin Y., Gong M., Zhang J., Li N., Na Y. (2015). Fungus ball and emphysematous cystitis secondary to Candida tropicalis: A case report. Can. Urol. Assoc. J..

[286] Garg V. (2015). Comparison of Clinical Presentation and Risk Factors in Diabetic and Non- Diabetic Females with Urinary Tract Infection Assessed as Per the European Association of Urology Classification. J. Clin. Diagnostic Res..

[287] Suzuki M., Hiramatsu M., Fukazawa M., Matsumoto M., Honda K., Suzuki Y., Kawabe Y. (2014). Effect of SGLT2 inhibitors in a murine model of urinary tract infection with Candida albicans. Diabetes Obes. Metab..

[288] Tumbarello M., Posteraro B., Trecarichi E., Al E. (2007). Biofilm production by Candida species and inadequate antifungal therapy as predictors of mortality for patients with candidemia. J. Clin. Microbiol..

[289] Michalopoulos A., Kriaras J., Geroulanos S. (1997). Systemic candidiasis in cardiac surgery patients. Eur. J. Cardiothorac. Surg..

[290] Sievert D.M., Ricks P., Edwards J.R., Schneider A., Patel J., Srinivasan A., Kallen A., Limbago B., Fridkin S., National Healthcare Safety Network (NHSN) Team and Participating NHSN Facilities (2013). Antimicrobial-Resistant Pathogens Associated with HealthcareAssociated Infections: Summary of Data Reported to the National Healthcare Safety Network at the Centers for Disease Control and Prevention, 2009–2010. Infect. Control Hosp. Epidemiol..

[291] Muskett H., Shahin J., Eyres G., Harvey S., Rowan K., Harrison D. (2011). Risk factors for invasive fungal disease in critically ill adult patients: A systematic review. Crit. Care.

[292] Paphitou N.I., Ostrosky-Zeichner L., Rex J.H. (2005). Rules for identifying patients at increased risk for candidal infections in the surgical intensive care unit: Approach to developing practical criteria for systematic use in antifungal prophylaxis trials. Med. Mycol..

[293] Michalopoulos A.S., Geroulanos S., Mentzelopoulos S.D. (2003). Determinants of Candidemia and Candidemia-Related Death in Cardiothoracic ICU Patients. Clin. Investig. Crit. Care.

[294] Wu J.-Q., Zhu L.-P., Ou X.-T., Xu B., Hu X.-P., Wang X., Weng X.-H. (2010). Epidemiology and risk factors for non- *Candida albicans* candidemia in non-neutropenic patients at a Chinese teaching hospital. Med. Mycol..

[295] Pfaller M., Jones R., Doern G., Fluit A., Verhoef J., Sader H., Messer S., Houston A., Coffman S., Hollis R. (1999). International surveillance of blood stream infections due to Candida species in the European SENTRY Program: Species distribution and antifungal susceptibility including the investigational triazole and echinocandin agents. SENTRY Participant Group (Euro). Diagn. Microbiol. Infect. Dis..

[296] Shekari Ebrahim Abad H., Zaini F., Kordbacheh P., Mahmoudi M., Safara M., Mortezaee V. (2015). In Vitro Activity of Caspofungin Against Fluconazole-Resistant Candida Species Isolated From Clinical Samples in Iran. Jundishapur J. Microbiol..

[297] Wu Z., Liu Y., Feng X., Liu Y., Wang S., Zhu X., Chen Q., Pan S. (2014). Candidemia: Incidence rates, type of species, and risk factors at a tertiary care academic hospital in China. Int. J. Infect. Dis..

[298] Barchiesi F., Spreghini E., Tomassetti S., Della Vittoria A., Arzeni D., Manso E., Scalise G. (2006). Effects of caspofungin against Candida guilliermondii and Candida parapsilosis. Antimicrob. Agents Chemother..

[299] Zepelin M.B.-V., Kunz L., Ruchel R., Reichard U., Weig M., Gross U. (2007). Epidemiology and antifungal susceptibilities of Candida spp. to six antifungal agents: Results from a surveillance study on fungaemia in Germany from July 2004 to August 2005. J. Antimicrob. Chemother..

[300] Golden S.H., Peart-Vigilance C., Kao W.H., Brancati F.L. (1999). Perioperative glycemic control and the risk of infectious complications in a cohort of adults with diabetes. Diabetes Care.

[301] Desnos-Ollivier M., Ragon M., Robert V., Raoux D., Gantier J.-C., Dromer F. (2008). Debaryomyces hansenii (Candida famata), a Rare Human Fungal Pathogen Often Misidentified as Pichia guilliermondii (Candida guilliermondii). J. Clin. Microbiol..

[302] Savini V., Catavitello C., Di Marzio I., Masciarelli G., Astolfi D., Balbinot A., Bianco A., Pompilio A., Di Bonaventura G., D’Amario C. (2010). Pan-azole-Resistant Candida guilliermondii from a Leukemia Patient’s Silent Funguria. Mycopathologia.

[303] Savini V., Catavitello C., Onofrillo D., Masciarelli G., Astolfi D., Balbinot A., Febbo F., D’Amario C., D’Antonio D. (2011). What do we know about Candida guilliermondii? A voyage throughout past and current literature about this emerging yeast. Mycoses.

[304] Hamilton H.C., Foxcroft D., Hamilton H.C. (2007). Central venous access sites for the prevention of venous thrombosis, stenosis and infection in patients requiring long-term intravenous therapy. Cochrane Database of Systematic Reviews.

[305] Ma X., Sun W., Liu T. (2006). [Clinical characteristics of Candida septicemia seen in a neonatal intensive care unit: Analysis of 9 cases]. Zhonghua er ke za zhi = Chin. J. Pediatr..

[306] Patel G.P., Simon D., Scheetz M., Crank C.W., Lodise T., Patel N. (2009). The Effect of Time to Antifungal Therapy on Mortality in Candidemia Associated Septic Shock. Am. J. Ther..

[307] Colombo A.L., Nucci M., Park B.J., Nouér S.A., Arthington-Skaggs B., da Matta D.A., Warnock D., Morgan J., Brazilian Network Candidemia Study, for the B.N.C. (2006). Epidemiology of candidemia in Brazil: A nationwide sentinel surveillance of candidemia in eleven medical centers. J. Clin. Microbiol..

[308] Horn D.L., Neofytos D., Anaissie E.J., Fishman J.A., Steinbach W.J., Olyaei A.J., Marr K.A., Pfaller M.A., Chang C., Webster K.M. (2009). Epidemiology and Outcomes of Candidemia in 2019 Patients: Data from the Prospective Antifungal Therapy Alliance Registry. Clin. Infect. Dis..

[309] Pfaller M.A., Diekema D.J. (2007). Epidemiology of invasive candidiasis: A persistent public health problem. Clin. Microbiol. Rev..

[310] Nucci M., Queiroz-Telles F., Tobón A.M., Restrepo A., Colombo A.L. (2010). Epidemiology of Opportunistic Fungal Infections in Latin America. Clin. Infect. Dis..

[311] Ryan T., Mc Carthy J.F., Rady M.Y., Serkey J., Gordon S., Starr N.J., Cosgrove D.M. (1997). Early bloodstream infection after cardiopulmonary bypass: Frequency rate, risk factors, and implications. Crit. Care Med..

[312] Leroy O., Gangneux J.-P., Montravers P., Mira J.-P., Gouin F., Sollet J.-P., Carlet J., Reynes J., Rosenheim M., Regnier B. (2009). Epidemiology, management, and risk factors for death of invasive Candida infections in critical care: A multicenter, prospective, observational study in France (2005–2006). Crit. Care Med..

[313] Shorr A.F., Lazarus D.R., Sherner J.H., Jackson W.L., Morrel M., Fraser V.J., Kollef M.H. (2007). Do clinical features allow for accurate prediction of fungal pathogenesis in bloodstream infections? Potential implications of the increasing prevalence of non-albicans candidemia. Crit. Care Med..

[314] Corzo-leon D.E., Alvarado-matute T., Colombo A.L., Cornejo-juarez P., Cortes J., Echevarria J.I., Macias A.E., Nucci M., Ostrosky-Zeichner L., Ponce-de-Leon A. (2014). Surveillance of Candida spp Bloodstream Infections: Epidemiological Trends and Risk Factors of Death in Two Mexican Tertiary Care Hospitals. PLoS ONE.

[315] Nucci M., Queiroz-Telles F., Alvarado-Matute T., Tiraboschi I.N., Cortes J., Zurita J., Guzman-Blanco M., Santolaya M.E., Thompson L., Sifuentes-Osornio J. (2013). Epidemiology of Candidemia in Latin America: A Laboratory-Based Survey. PLoS ONE.

[316] Gupta A., Gupta A., Varma A. (2015). Candida glabrata candidemia: An emerging threat in critically ill patients. Indian J. Crit. Care Med..

[317] Pozzilli P., Leslie R.D. (1994). Infections and diabetes: Mechanisms and prospects for prevention. Diabet. Med..

[318] MacCuish A.C., Urbaniak S.J., Campbell C.J., Duncan L.J., Irvine W.J. (1974). Phytohemagglutinin transformation and circulating lymphocyte subpopulations in insulin-dependent diabetic patients. Diabetes.

[319] Hostetter M.K. (1990). Handicaps to host defense. Effects of hyperglycemia on C3 and Candida albicans. Diabetes.

[320] Padawer D., Pastukh N., Nitzan O., Labay K., Aharon I., Brodsky D., Glyatman T., Peretz A. (2015). Catheter-associated candiduria: Risk factors, medical interventions, and antifungal susceptibility. Am. J. Infect. Control.

[321] Tambyah P.A., Halvorson K.T., Maki D.G. (1999). A Prospective Study of Pathogenesis of Catheter-Associated Urinary Tract Infections. Mayo Clin. Proc..

[322] Maki D.G., Tambyah P.A. (2001). Engineering out the risk for infection with urinary catheters. Emerg. Infect. Dis..

[323] Kuhn D.M., Mikherjee P.K., Clark T.A., Pujol C., Chandra J., Hajjeh R.A., Warnock D.W., Soil D.R., Ghannoum M.A. (2004). Candida parapsilosis characterization in an outbreak setting. Emerg. Infect. Dis..

[324] Khatib R., Johnson L.B., Fakih M.G., Riederer K., Briski L. (2016). Current trends in candidemia and species distribution among adults: *Candida glabrata* surpasses *C. albicans* in diabetic patients and abdominal sources. Mycoses.

[325] Lipsett P.A. (2006). Surgical critical care: Fungal infections in surgical patients. Crit. Care Med..

[326] Hattori H., Maeda M., Nagatomo Y., Takuma T., Niki Y., Naito Y., Sasaki T., Ishino K. (2018). Epidemiology and risk factors for mortality in bloodstream infections: A single-center retrospective study in Japan. Am. J. Infect. Control.

[327] Zhao S.J., Fu Y.Q., Zhu M.X., Zhou H., Xu M., Yan R.L., Shui Y.X., Zhou J.Y. (2017). [Patients of Escherichia coli bloodstream infection: Analysis of antibiotic resistance and predictors of mortality]. Zhonghua Yi Xue Za Zhi.

[328] Tumbarello M., Fiori B., Trecarichi E.M., Posteraro P., Losito A.R., de Luca A., Sanguinetti M., Fadda G., Cauda R., Posteraro B. (2012). Risk factors and outcomes of candidemia caused by biofilm-forming isolates in a tertiary care hospital. PLoS ONE.

[329] Bristow I.R., Spruce M.C. (2009). Fungal foot infection, cellulitis and diabetes: A review. Diabet. Med..

[330] Tchernev G., Penev P.K., Nenoff P., Zisova L.G., Cardoso J.C., Taneva T., Ginter-Hanselmayer G., Ananiev J., Gulubova M., Hristova R. (2013). Onychomycosis: Modern diagnostic and treatment approaches. Wiener Medizinische Wochenschrift.

[331] Chang S.-J., Hsu S.-C., Tien K.-J., Hsiao J.-Y., Lin S.-R., Chen H.-C., Hsieh M.-C. (2008). Metabolic syndrome associated with toenail onychomycosis in Taiwanese with diabetes mellitus. Int. J. Dermatol..

[332] Kumar D., Banerjee T., Chakravarty J., Singh S., Dwivedi A., Tilak R. (2016). Identification, antifungal resistance profile, in vitro biofilm formation and ultrastructural characteristics of Candida species isolated from diabetic foot patients in Northern India. Indian J. Med. Microbiol..

[333] Raiesi O., Siavash M., Mohammadi F., Chabavizadeh J., Mahaki B., Maherolnaghsh M., Dehghan P. (2017). Frequency of Cutaneous Fungal Infections and Azole Resistance of the Isolates in Patients with Diabetes Mellitus. Adv. Biomed. Res..

[334] Lugo-Somolinos A., Sánchez J.L. (1992). Prevalence of dermatophytosis in patients with diabetes. J. Am. Acad. Dermatol..

[335] Pierard G.E., Pierard-Franchimont C. (2005). The nail under fungal siege in patients with type II diabetes mellitus. Mycoses.

[336] Dogra S., Kumar B., Bhansali A., Chakrabarty A. (2002). Epidemiology of onychomycosis in patients with diabetes mellitus in India. Int. J. Dermatol..

[337] Eckhard M., Lengler A., Liersch J., Bretzel R.G., Mayser P. (2007). Fungal foot infections in patients with diabetes mellitus-results of two independent investigations. Mycoses.

[338] Wijesuriya T.M., Weerasekera M.M., Kottahachchi J., Ranasinghe K.N.P., Dissanayake M.S.S., Prathapan S., Gunasekara T.D.C.P., Nagahawatte A., Guruge L.D., Bulugahapitiya U. (2014). Proportion of lower limb fungal foot infections in patients with type 2 diabetes at a tertiary care hospital in Sri Lanka. Indian J. Endocrinol. Metab..

[339] Boyko E.J., Ahroni J.H., Cohen V., Nelson K.M., Heagerty P.J. (2006). Prediction of Diabetic Foot Ulcer Occurrence Using Commonly Available Clinical Information: The Seattle Diabetic Foot Study. Diabetes Care.

[340] Johargy A.K. (2016). Antimicrobial susceptibility of bacterial and fungal infections among infected diabetic patients. J. Pak. Med. Assoc..

[341] Gupta A.K., Konnikov N., Macdonald P., Rich P., Rodger N.W., Edmonds M.W., Mcmanus R., Summerbell R.C. (1998). Prevalence and epidemiology of toenail onychomycosis in diabetic subjects: A multicentre survey. Br. J. Dermatol..

[342] Chi C.-C., Wang S.-H., Chou M.-C. (2005). The causative pathogens of onychomycosis in southern Taiwan. Mycoses.

[343] Wang D., Jiang Y., Li Z., Xue L., Li X., Liu Y., Li C., Wang H. (2018). The Effect of Candida albicans on the Expression Levels of Toll-like Receptor 2 and Interleukin-8 in HaCaT Cells Under High- and Low-glucose Conditions. Indian J. Dermatol..

[344] Delamaire M., Maugendre D., Moreno M., Le Goff M.-C., Allannic H., Genetet B. (1997). Impaired Leucocyte Functions in Diabetic Patients. Diabet. Med..

[345] Llorente L., De La Fuente H., Richaud-Patin Y., Alvarado-De La Barrera C., Diaz-Borjón A., López-Ponce A., Lerman-Garber I., Jakez-Ocampo J. (2000). Innate immune response mechanisms in non-insulin dependent diabetes mellitus patients assessed by flow cytoenzymology. Immunol. Lett..

[346] De Souza Ferreira C., Pennacchi P.C., Araújo T.H., Taniwaki N.N., De Araújo Paula F.B., Da Silveira Duarte S.M., Rodrigues M.R. (2016). Aminoguanidine treatment increased NOX2 response in diabetic rats: Improved phagocytosis and killing of Candida albicans by neutrophils. Eur. J. Pharmacol..

[347] Pupim A.C.E., Campois T.G., Araújo E.J.A., Svidizinski T.I.E., Felipe I. (2017). Infection and tissue repair of experimental cutaneous candidiasis in diabetic mice. J. Med. Microbiol..

[348] Rubin B.G., German M.L., Louis S. (1994). Candida infection with aneurysm formation in the juxtarenal aorta. J. Vasc. Surg..

[349] King A.J.F. (2012). The use of animal models in diabetes research. Br. J. Pharmacol..

[350] Brosius F.C., Alpers C.E., Bottinger E.P., Breyer M.D., Coffman T.M., Gurley S.B., Harris R.C., Kakoki M., Kretzler M., Leiter E.H. (2009). Mouse Models of Diabetic Nephropathy. J. Am. Soc. Nephrol..

[351] Sullivan K.A., Hayes J.M., Wiggin T.D., Backus C., Su Oh S., Lentz S.I., Brosius F., Feldman E.L. (2007). Mouse models of diabetic neuropathy. Neurobiol. Dis..

[352] Sullivan K.A., Lentz S.I., Roberts J.L., Feldman E.L. (2008). Criteria for Creating and Assessing Mouse Models of Diabetic Neuropathy. Curr. Drug Targets.

[353] Franconi F., Seghieri G., Canu S., Straface E., Campesi I., Malorni W. (2008). Are the available experimental models of type 2 diabetes appropriate for a gender perspective?. Pharmacol. Res..

[354] Inada A., Arai H., Nagai K., Miyazaki J., Yamada Y., Seino Y., Fukatsu A. (2007). Gender Difference in ICER Iγ Transgenic Diabetic Mouse. Biosci. Biotechnol. Biochem..

[355] Hassan A., Poon W., Baker M., Linton C., Mühlschlegel F.A. (2012). Confirmed Candida albicans endogenous fungal endophthalmitis in a patient with chronic candidiasis. Med. Mycol. Case Rep..

[356] Woodrum D.T., Welke K.F., Fillinger M.F. (2001). Candida infection associated with a solitary mycotic common iliac artery aneurysm. J. Vasc. Surg..

[357] Dowling R.D., Baladi N., Zenati M., Dummer J.S., Kormos R.L., Armitage J.M., Yousem S.A., Hardesty R.L., Griffith B.P. (1990). Disruption of the aortic anastomosis after heart-lung transplantation. Ann. Thorac. Surg..

[358] Laouad I., Buchler M., Noel C., Sadek T., Maazouz H., Westeel P.F., Lebranchu Y. (2005). Renal Artery Aneurysm Secondary to Candida albicans in Four Kidney Allograft Recipients. Transplant. Proc..

[359] Mai H., Champion L., Ouali N., Hertig A., Peraldi M.-N., Glotz D., Rondeau E., Costa M.-A., Snanoudj R., Benoit G. (2006). Candida albicans Arteritis Transmitted by Conservative Liquid After Renal Transplantation: A Report of Four Cases and Review of the Literature. Transplantation.

[360] Valentine R.J., Chung J. (2012). Primary Vascular Infection. Curr. Probl. Surg..

[361] Chapuis-Taillard C., Manuel O., Bille J., Calandra T., Rotman S., Tarr P.E. (2008). *Candida* Arteritis in Patients Who Have Not Received Organ Transplants: Case Report and Review of the Literature. Clin. Infect. Dis..

[362] Brown S.L., Busuttil R.W., Baker J.D., Machleder H.I., Moore W.S., Barker W.F. (1984). Bacteriologic and surgical determinants of survival in patients with mycotic aneurysms. J. Vasc. Surg..

[363] Potti A., Danielson B., Sen K. (1998). “True” mycotic aneurysm of a renal artery allograft. Am. J. Kidney Dis..

[364] Oderich G.S., Panneton J.M., Bower T.C., Cherry K.J., Rowland C.M., Noel A.A., Hallett J.W., Gloviczk P. (2001). Infected aortic aneurysms: Aggressive presentation, complicated early outcome, but durable results. J. Vasc. Surg..

[365] Sergio P., De Araujo R., Medeiros Z., Melo F.L. (2013). De Case Report Candida famata- induced fulminating cholecystitis. Rev Soc Bras Med Trop..

[366] Kakeya H., Izumikawa K., Yamada K., Narita Y., Nishino T., Obata Y., Takazono T., Kurihara S., Kosai K., Morinaga Y. (2014). Concurrent subcutaneous candidal abscesses and pulmonary cryptococcosis in a patient with diabetes mellitus and a history of corticosteroid therapy. Intern Med..

[367] Florescu D.F., Brostrom S.E., Dumitru I., Kalil A.C. (2010). Candida albicans Skin Abscess in a Heart Transplant Recipient. Infect. Dis. Clin. Pract..

[368] Neves N., Santos L., Reis C., Sarmento A. (2014). Candida albicans brain abscesses in an injection drug user patient: A case report. BMC Res. Notes.

[369] Honda H., Warren D.K. (2009). Central Nervous System Infections: Meningitis and Brain Abscess. Infect. Dis. Clin. N. Am..

[370] Fennelly A.M., Slenker A.K., Murphy L.C., Moussouttas M., DeSimone J.A. (2013). *Candida* cerebral abscesses: A case report and review of the literature. Med. Mycol..

[371] Yang C.-H., He X.-S., Chen J., Ouyang B., Zhu X.-F., Chen M.-Y., Xie W.-F., Chen L., Zheng D.-H., Zhong Y. (2012). Fungal infection in patients after liver transplantation in years 2003 to 2012. Ann. Transplant..

[372] Abraham G., Kumar V., Nayak K.S., Ravichandran R., Srinivasan G., Krishnamurthy M., Prasath A.K., Kumar S., Thiagarajan T., Mathew M. (2010). Predictors of long-term survival on peritoneal dialysis in south india: A multicenter study. Perit. Dial. Int..

[373] Yuvaraj A., Rohit A., Koshy P.J., Nagarajan P., Nair S., Abraham G. (2014). Rare occurrence of fatal Candida haemulonii peritonitis in a diabetic CAPD patient. Ren. Fail..

[374] Zappella N., Desmard M., Chochillon C., Ribeiro-Parenti L., Houze S., Marmuse J.P., Montravers P. (2015). Positive peritoneal fluid fungal cultures in postoperative peritonitis after bariatric surgery. Clin. Microbiol. Infect..

[375] Mulcahy J.J. (2000). Long-term experience with salvage of infected penile implants. J. Urol..

[376] Peppas D.S., Moul J.W., McLeod D.G. (1988). Candida albicans corpora abscess following penile prosthesis placement. J. Urol..

[377] Mulcahy J.J., Carson C.C. (2011). Long-Term Infection Rates in Diabetic Patients Implanted With Antibiotic-Impregnated Versus Nonimpregnated Inflatable Penile Prostheses: 7-Year Outcomes. Eur. Urol..

[378] Cotta B.H., Butcher M., Welliver C., Mcvary K., Köhler T. (2015). Two Fungal Infections of Inflatable Penile Prostheses in Diabetics. Sex. Med..

[379] Maatouk I., Hajjar M., Moutran R. (2015). Candida albicans and Streptococcus pyogenes balanitis: Diabetes or STI?. Int. J. Std Aids.

[380] Wróblewska M., Kuzaka B., Borkowski T., Kuzaka P., Kawecki D., Radziszewski P. (2014). Fournier’s Gangrene—Current Concepts. Polish J. Microbiol..

[381] Saha K., Sit N.K., Maji A., Jash D. (2013). Recovery of fluconazole sensitive Candida ciferrii in a diabetic chronic obstructive pulmonary disease patient presenting with pneumonia. Lung India.

